# Synthesis of Trichodermin Derivatives and Their Antimicrobial and Cytotoxic Activities

**DOI:** 10.3390/molecules24203811

**Published:** 2019-10-22

**Authors:** Javier E. Barúa, Mercedes de la Cruz, Nuria de Pedro, Bastien Cautain, Rosa Hermosa, Rosa E. Cardoza, Santiago Gutiérrez, Enrique Monte, Francisca Vicente, Isidro G. Collado

**Affiliations:** 1 Biological Chemistry Department, Chemistry Science Faculty, National University of Asunción, Ruta Mcal. Estigarribia Km 11.5, San Lorenzo 2160, Paraguay; javierbarua@qui.una.py; 2 Department of Organic Chemistry, Campus of Puerto Real, Science Faculty, University of Cádiz, 11510 Puerto Real, Cádiz, Spain; 3 Fundación Medina, Avda. del Conocimiento 34, Armilla, 18016 Granada, Spain; mercedes.delacruz@medinaandalucia.es (M.d.l.C.); nuri_dp@hotmail.es (N.d.P.); bastien.cautain@medinaandalucia.es (B.C.); francisca.vicente@medinaandalucia.es (F.V.); 4 Department of Microbiology and Genetics, Spanish-Portuguese Institute for Agricultural Research (CIALE), University of Salamanca, Campus of Villamayor, Rio Duero 12, 37185 Salamanca, Spain; rhp@usal.es (R.H.); emv@usal.es (E.M.); 5 University of León, Campus of Ponferrada, Superior and Technical Universitary School of Agricultural Engineers, Area of Microbiology, Avda. Astorga s/n, 24400 Ponferrada, Spain; re.cardoza@unileon.es (R.E.C.); s.gutierrez@unileon.es (S.G.)

**Keywords:** trichothecene, sesquiterpenes, *tri* genes, synthesis, antimicrobial, cytotoxic bioactivity

## Abstract

Trichothecene mycotoxins are recognized as highly bioactive compounds that can be used in the design of new useful bioactive molecules. In *Trichoderma brevicompactum*, the first specific step in trichothecene biosynthesis is carried out by a terpene cyclase, trichodiene synthase, that catalyzes the conversion of farnesyl diphosphate to trichodiene and is encoded by the *tri5* gene. Overexpression of *tri5* resulted in increased levels of trichodermin, a trichothecene-type toxin, which is a valuable tool in preparing new molecules with a trichothecene skeleton. In this work, we developed the hemisynthesis of trichodermin and trichodermol derivatives in order to evaluate their antimicrobial and cytotoxic activities and to study the chemo-modulation of their bioactivity. Some derivatives with a short chain at the C-4 position displayed selective antimicrobial activity against *Candida albicans* and they showed MIC values similar to those displayed by trichodermin. It is important to highlight the cytotoxic selectivity observed for compounds **9**, **13**, and **15**, which presented average IC_50_ values of 2 μg/mL and were cytotoxic against tumorigenic cell line MCF-7 (breast carcinoma) and not against Fa2N4 (non-tumoral immortalized human hepatocytes).

## 1. Introduction

Natural products (NPs, secondary metabolites) are an invaluable source of inspiration in drug design and development. Having evolved over several millennia to acquire specific ligand–protein binding motifs, their structures cover a wide range of biologically relevant chemical space that cannot be efficiently explored by synthetic compounds in commercially available screening libraries [1,2]. Of all new drugs approved between 1981 and 2014 (*n* = 1562), 50% are natural products, are derived from natural products, or are synthetic molecules inspired by or that mimic natural products [3]. Terpenoids are the largest class of NPs, with about 60% of NP diversity originating from the terpene pathway [4]. All terpenes are derived from the repetitive fusion of branched five-carbon units based on an isopentene skeleton, and most of the chemical intermediates in their biosynthetic pathway are known [5]. Typical structures contain carbon skeletons represented by (**C5**)n and are classified as hemiterpenes (**C5**), monoterpenes (**C10**), sesquiterpenes (**C15**), diterpenes (**C20**), sesterterpenes (**C25**), triterpenes (**C30**), and tetraterpenes (**C40**) [6].

Trichothecenes are a particular type of sesquiterpene which are a family of metabolites that have mainly been isolated from species of *Fusarium* and certain other fungal genera like *Cephalosporium*, *Isaria*, *Microcyclospora*, *Myrothecium*, *Spicellum*, *Stachybotrys*, *Trichoderma*, and *Trichothecium*. The biosynthesis of trichothecenes is well documented and has been the focus of several detailed reviews [7,8]. All trichothecenes contain a common skeleton or tricyclic ring that typically contains an epoxide between the C-12 and C-13 carbons and a varying number of hydroxyl or acetoxy groups [9]. This sesquiterpene family exhibits cytotoxic activity against different eukaryotes cell lines by induction of apoptosis [10], and also acts as a fungicide [11], an immunosuppressant [12] and a neurotoxin [13].

The biosynthetic pathway of trichothecenes has been widely studied in *Fusarium* species [14] and has recently been described in *Trichoderma* species [15,16]. In both cases, the genes involved are organized in clusters. The cyclization of farnesyl diphosphate to trichodiene, the first step in the biosynthetic pathway of approximately 150 different toxic trichothecenes, is catalyzed by the enzyme trichodiene synthase which is encoded by the *tri5* gene. This *tri5* gene has been isolated and characterized in *Trichoderma brevicompactum*, the only *Trichoderma* species that is capable of producing trichodermin (**1**) [17] and overexpression of the *tri5* gene has shown that it is involved in the production of **1** and in the antifungal activity exhibited by *T. brevicompactum* [5].

Trichodermin (**1**) is a member of a family of sesquiterpene metabolites that possesses an olefinic group at positions C-9 and C-10 and an epoxide group between C-12 and C-13, on a trichothecene skeleton, characterizing it as a 12,13-epoxytrichothecene [18]. This compound diffuses rapidly through the cell membrane and binds to the eukaryotic ribosome to inhibit the translation of proteins by means of interaction with the peptidyl transferase [19]. It has been shown that trichodermin (**1**) is a potent inhibitor of protein synthesis in mammals and its inhibitory activity requires the presence of both the C9-C10 olefinic group and the characteristic C12-C13 epoxide group [9]. Trichodermin (**1**) has also exhibited potent antimicrobial activity against filamentous fungi and yeasts and bacteria [5]. In these studies, the overexpression of the *tri5* gene resulted in an increase of the level of transcription of three other trichothecene genes, *tri4*, *tri6*, and *tri10* [20].

In view of the broad biological activity shown and the pharmacological potential inherent in the chemical structure of **1**, this work deals with the synthesis of trichodermin derivatives to study the potential chemo-modulation of bioactivity and to evaluate their antimicrobial and cytotoxic activity against a panel of prokaryotic and eukaryotic organisms and different types of cellular lines, respectively, through an analysis of the relationship between the chemical structure and activity of these derivatives.

## 2. Results and Discussion

### 2.1. Synthesis of Trichodermin Derivatives

The potency and broad biological activity shown by trichodermin (**1**) sparked our interest in amassing adequate amounts of this molecule to complete a study of its biological activity and to perform chemical transformations on the **1** skeleton with a view to modulating or enhancing this biological activity. Several efforts have been made to modulate the activity of trichothecenes as antifungal agents [21,22,23] with promising results. Total synthesis of trichodermin (**1**) [24] and trichodermol (**2**) [25,26] has been proposed. However, this involves several reaction steps, making it expensive and inefficient.

In recent years, we have worked on obtaining transformants of *T. brevicompactum* in order to study the functionality of the genes involved in the biosynthesis of trichothecenes and to obtain mutants to efficiently produce large quantities of trichodermin (**1**) or trichodermol (**2**), the final precursor of **1** in its biosynthetic pathway. To that end, our group has analyzed the production of trichothecenes by transformants overexpressing the *tri5* gene. We found that trichodermin (**1**) production in these strains increased dramatically compared to that of the parent strain, achieving yields up to 32.3% of dry crude extract compared to the wild strain that reached levels of 11.3 mg/mL after 14 days of fermentation [5]. 

With this knowledge, and to obtain adequate amounts of trichodermin (**1**) needed for the synthesis of its derivatives, we fermented *T. brevicompactum* IBT40841-derived *tri5*-overexpressing transformant Tb41tri5 [5] in PDB medium for 14 days, which produced, by extraction of the fermentation broth with ethyl acetate, 1.4 g of crude extract. This was preliminarily analyzed by TLC and ^1^H-NMR. From the chromatographic separation of the crude extract, a compound **1**–enriched fraction was obtained, which was then purified by HPLC and characterized by extensive NMR experiments to yield 815 mg of the pure compound.

**Scheme 1:**
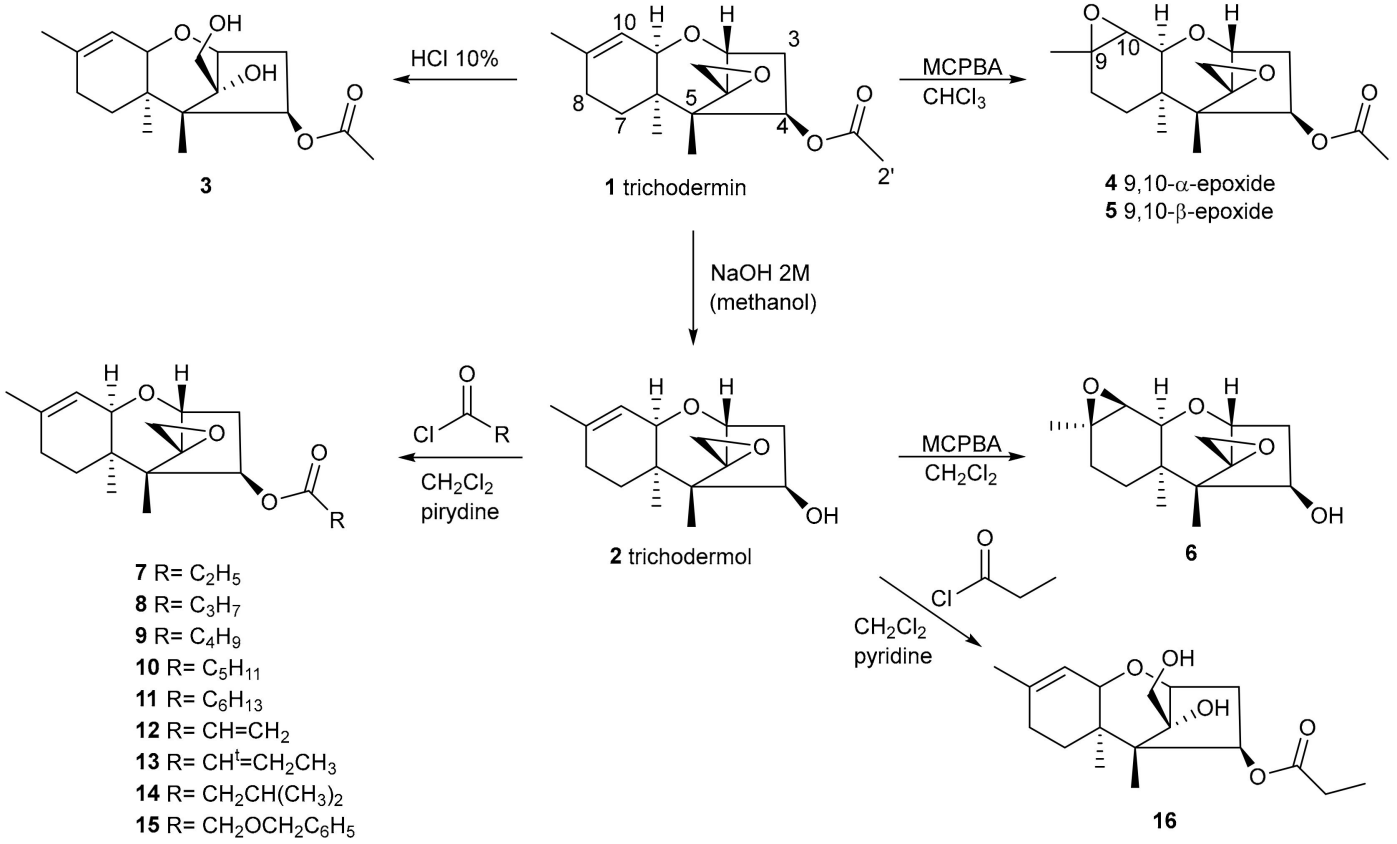
Chemical transformations carried out on trichodermin (**1**) and trichodermol (**2**) for the preparation of compounds **3**–**16**.

Scheme 1 shows the chemical transformations performed on **1**. Modifications were made on the olefin located on the C9–C10 carbons and on the C12–C13 epoxide. Furthermore, a group of nine compounds with different substituents at the C4 carbon were prepared by esterifying the hydroxyl group at this position. All the products obtained were purified by HPLC, and their structures were elucidated by spectroscopic techniques.

Product **3** was prepared at a yield of 49% by treating **1** with a stirred aqueous solution of HCl (10%) for 15 h. Its molecular formula was confirmed by High Resolution Mass Spectrometry (HRMS) where an *m/z* ion [C_17_H_26_O_5_ + H]^+^ 311.1837 was observed. Furthermore, the opening of the epoxide at C12-C13 of **1** was revealed by the signals in its ^1^H-NMR spectrum at *δ* 3.88 ppm, which integrated for 2H corresponding to the two H-13 protons. 

Products **4** and **5** were prepared by treating **1**, dissolved in chloroform, with *m*-chloroperbenzoic acid (MCPBA). The reaction yielded two epoxidation products corresponding to C9-C-10-α-epoxide (**4**) and the epoxide with β orientation (**5**), with yields of 7.2% and 82.3%, respectively. The loss of the signals corresponding to the C9-C-10 olefin of trichodermin (**1**) was confirmed by means of ^1^H-NMR and ^13^C-NMR spectroscopy. HRMS data confirmed the expected molecular formula C_17_H_24_O_5_ for both products. Lastly, stereochemistry was assigned based on observed nuclear Overhauser effect (NOE) interactions. Thus, in NOESY-1D experiments performed on **5**, irradiation of H-10 produced enhancements at H-11, H-15, and H-16, determining the β orientation of the epoxide for this derivative.

Trichodermol (**2**) was obtained from the alkaline treatment of **1** by stirring in a methanolic solution of NaOH (2M). The reaction product **2** was confirmed by comparing its NMR signals with those described in the literature [27]. The loss of the acetyl group at position C-4 left 4-OH free, an easily accessible function for the introduction of various substituents. This product was used to prepare derivatives **6**–**16**.

Product **6** was prepared from trichodermol (**2**) by means of an epoxidation reaction similar to the one described for **4** and **5**. All its physical constants were concordant with the proposed structure. 

Products **7**–**15** were prepared from the esterification of the hydroxyl group at C-4 of compound **2**, with the corresponding acyl chlorides in the presence of pyridine. Good yields of all the products were obtained, and their spectroscopic data coincided with the proposed structures. Product **16** was obtained from a reaction of **2** with an excess of propanoyl chloride in the presence of pyridine. 

The acid medium, probably due to a large excess of the reagent, led to the opening of the 12,13-epoxide of **2**, simultaneously with the introduction of a propanoyl group at position C-4. The mass spectrum showed an ion *m/z* 347.1830 (calculated for C_18_H_28_O_5_Na [M + Na]^+^ 347.1834), verifying the expected molecular formula.

### 2.2. Evaluation of the Cytotoxic and Antimicrobial Activity of Trichodermin (***1***) Derivatives

Antimicrobial and cytotoxic activity tests were performed against a panel of microorganisms and human cell lines to evaluate the biological activity of trichodermin (**1**), trichodermol (**2**), and derivatives **3**–**16**, respectively. Table 1 summarizes the results of the antimicrobial activity expressed as Minimum Inhibitory Concentrations (MIC). None of the compounds showed significant activity against *Acinetobacter baumannii*, *Pseudomonas aeruginosa*, *Staphylococcus aureus*, or *Bacillus subtilis*. These results are presented as Supplementary Materials (Table S2). Compounds **9**–**11** mildly inhibited the growth of *Escherichia coli* 5746.

However, as was expected [5], trichodermin (**1**) exhibited high inhibition against the yeast *Candida albicans* (<4 μg/mL). Compounds **2**, **7**, and **14** exhibited moderate activity (8–32 μg/mL), while the activity of compounds **9**, **10**, and **15** can be considered interesting with values between 4–8 μg/mL. Special mention should be made of the activity of compounds **12** and **13** with values of 4 μg/mL, the most active of the series except for trichodermin (**1**). Compounds **9** and **12** were previously evaluated in antifungal assays against *Botrytis cinerea, Peronophythora litchii, Rhizoctonia solani, Sclerotonia sclerotiorum*, and *Magnaporthe grisea* [21]. 

In other studies, compound **10** exhibited good antifungal activity against *Ustilaginoidea virens*, *R. solani*, and *M. grisea* [22].

The specificity of the activity of the compounds herein described against *C. albicans* should be highlighted. They exhibited good inhibition activity against this yeast and low or no activity against the bacterial groups. A comparison of activities and chemical structures showed that the hydroxyl at C-4 must be esterified with a short-chain ester. However, the presence of a double bond at C-2′ in **12** and **13** produces an increase in biological activity close to **1**. Compounds **3**–**6** and **16** completely lost the inhibitory capacity of their precursor trichodermin (**1**), confirming that both the 12,13-epoxide group and the C9–C10 double bond play a fundamental role in the toxicity of these compounds [28].

Table 2 summarizes the results of the cytotoxic effect observed from trichothecene derivatives on different human cell lines. The results are presented as IC_50_ in μg/mL, measured after 24 h of incubation. None of the compounds showed significant cytotoxic activity against the A549 (lung carcinoma) or HT29 (colon carcinoma) lines after 24 h of incubation (Table S3). Trichodermin (**1**) showed intense cytotoxic activity against the MCF-7 line (breast carcinoma), and moderately inhibited RCC4-VA (renal carcinoma) and Fa2N4 (immortalized hepatocytes). Unfortunately, this compound exhibited little specificity in terms of its cytotoxic activity, acting indistinctly against both the carcinoma lines and the non-tumoral Fa2N4 line. It is well known that trichodermin is a very potent inhibitor of protein synthesis [29]. However, it is important to highlight the cytotoxicity of derivatives **9**, **10**, **12**, **13**, and **15** with approximate IC_50_ values of between 2–4 μg/mL. This can be considered promising as they were selective against the tumorigenic cell line MCF-7, without affecting the non-tumor line Fa2N4. 

A natural product called 8-deoxytrichothecin (**17**) was isolated from *Trichothecium roseum* [30] and *Spicellum roseum* [31,32] and induced the accumulation of glucosylceramide (GlcCer) and simultaneous reduction in the formation of lactosylceramide (LacCer) in complex gangliosides in primary cultured neurons [33]. It also significantly reduced the cell viabilities of HL-60, SMMC-7721, A549, MCF-7 and SW480 cell lines [34]. Compound **13** is an isomer of **17** with an E configuration in the double bond C2′–C3′. 
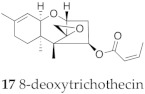


These results lead to the proposal of new chemical transformations that enhance the activities of these derivatives. Compared to antimicrobial activity results, the high toxicity of **1** against eukaryotic cells can be confirmed, with no noticeable effect on bacteria.

Compounds **3** and **16** lost all their activity in all the cell lines tested. These derivatives lost the 12,13-epoxide group of trichodermin (**1**), confirming that the presence of this group is key to the activity of these trichothecenes. Likewise, **5** is inactive under the conditions tested, demonstrating the importance of the C9-C10 double bond in the activity of these trichothecenes. Cell line MCF-7 proved to be especially sensitive to several of the compounds tested. Trichodermol (**2**) was less active compared to trichodermin (**1**) due to the loss of the acetyl group, although it still exhibited good activity. Derivatives **7**–**15**, obtained by the esterification of **2** at C4–OH, all have good IC_50_ values, especially compound **9** (4-pentanoyltrichodermol) with activity of 1.93 μg/mL, the most active of this series, followed by compounds **15** (2.03 μg/mL) and **13** (2.15 μg/mL). 

## 3. Experimental Section

### 3.1. General

Unless otherwise noted, materials and reagents were obtained from commercial suppliers and were used without further purification. Air- and moisture-sensitive reactions were performed under an argon atmosphere. Optical rotations were determined on a Perkin-Elmer 341 polarimeter (Perkin-Elmer, Seville, Spain). IR spectra were recorded on a Perkin-Elmer Spectrum BX FT-IR spectrophotometer (Perkin-Elmer, Seville, Spain). ^1^H and ^13^C-NMR spectra were obtained on a Varian INOVA 400 MHz NMR spectrometer (Varian, Palo Alto, CA, USA) using tetramethylsilane as an internal reference. NMR assignments were made using a combination of 1D and 2D techniques and, where appropriate, by comparison with assignments available in the literature for previously described compounds. High-Resolution Mass Spectroscopy (HRMS) was performed with a QTOF mass spectrometer in positive ion ESI or APCI (APGC^+^ for sample analyses using GC chromatography (Waters, Manchester, UK). HPLC was performed with a Hitachi/Merck L-6270 device equipped with a UV-VIS detector (L 4250) and a differential refractometer detector (RI-71) (VWR, Barcelona, Spain). TLC was performed on a Merck Kieselgel 60 F_254_, 0.2 mm thick, Silica gel (Merck KGaA, Darmstadt, Germany) was used for column chromatography. Purification by high-pressure liquid chromatography (HPLC) was performed using a Si gel column (LiChrospher Si 60, 10 μm, 1 cm wide, 25 cm long, (VWR, Barcelona, Spain).

### 3.2. Fungal Strain and Culture Conditions

*Trichoderma brevicompactum* IBT 40841 (=IBT40841) (IBT Culture Collection, Department of Biotechnology, Technical University of Denmarck, Kongens Lyngby, Denmark) and *tri5*-overexpressing transformant Tb41tri5 [5] were used throughout this study. This strain was maintained on potato dextrose agar medium (PDA) (Difco Becton Dickinson, Sparks, MD, USA).

Six micelial plugs (6 mm each) of Tb41tri5 transformant strain were used to inoculate 0.5-L flasks containing 250 mL of potato dextrose broth (PDB) (Difco Becton Dickinson). Each flask was cultured at 25 °C and 250 rpm in the dark for 14 days. Sixteen 0.5-L flasks were used for trichodermin (**1**) extraction.

The yeast *Candida albicans* MY1055 used as the target in antimicrobial assays was maintained on Sabouraud dextrose agar (SDA) (Difco Becton Dickinson).

### 3.3. Microorganism Used in the Bioassays

The following microorganisms were used in the antimicrobial bioassays: Methicillin-resistant *Staphylococcus aureus* MRSA 5393, *Staphylococcus aureus* EPI167, *Acinetobacter baumannii* MB5973, *Escherichia coli* MB2884 and MB5746, *Pseudomonas aeruginosa* MB5919, *Candida albicans* MY1055, and *Bacillus subtilis* MB964.

### 3.4. Chemical Procedures

#### 3.4.1. Trichodermin (**1**) Extraction

Four liters of fermentation broth were filtered through 200 μm Nylon filters and saturated with NaCl, and the aqueous phase was extracted with ethyl acetate (EtOAc). The EtOAc extract was washed three times with H_2_O and then dried over anhydrous Na_2_SO_4_. Evaporation of the solvent under reduced pressure afforded a dense oil that was separated by means of flash chromatography on silica gel, with a mixture of ethyl acetate/hexane (20% in EtOAc). The fractions were collected and purified by HPLC rendering 815 mg of pure trichodermin (**1**). Spectroscopic methods, specifically ^1^H NMR and ^13^C NMR, were then employed to identify the molecule. Spectroscopic data for compound **1** were identical to those described in the literature [18]. 

#### 3.4.2. Preparation of Compound **3**

An aqueous solution of hydrochloric acid (HCl) (5 mL, 10% *v*/*v*) was added to trichodermin (**1**) (20.0 mg, 0.068 mmol). The mixture was stirred overnight at room temperature, and when TLC analysis indicated completion of the reaction (14 h), a diluted solution of sodium hydroxide (NaOH) (1 M,) was added until pH = 7. The aqueous phase was extracted with ethyl acetate (3 × 20 mL), dried over anhydrous sodium sulfate and filtered. Evaporation of the solvent gave a crude product that was purified by silica gel column chromatography (petroleum ether/EtOAc, 80:20), to yield compound **3**.

*(4R,12S)-12-Hydroxy-12-(hydroxymethyl)trichothec-9-en-4-yl acetate* (**3**) [21] (10.3 mg, 49%). White solid; IR (film) *υ*_max_ 3461, 2968, 1734, 1238 cm^−1^; ^1^H-NMR (CDCl_3,_ 400 MHz) δ 5.50 (1H, m, H-10), 5.12 (1H, dd, *J* = 5.6 Hz, 10.0, H-4), 4.15 (1H, dd, *J* = 6.1, 11.3 Hz, H-2), 3.88 (1H, dd, *J* = 26.6, 7.3 Hz, H-13′), 3.88 (1H, dd, *J* = 7.3, 1.8 Hz, H-13′’), 3.75 (1H, d, *J* = 5.2 Hz, H-11), 2.57 (1H, dt, *J* = 5.8, 11.8, H-3), 2.14 (1H, t, OH-13), 2.05 (3H, s, H-2′), 1.96 (3H, m, H-3, H-8), 1.71 (1H, dd, H-7′), 1.71 (3H, s, H-16), 1.27 (1H, m, H-7′’), 1.16 (3H, s, H-14), 0.76 (3H, s, H-15); ^13^C NMR (CDCl_3_, 100 MHz) δ 170.2 (C-1′), 141.0 (C-9), 117.3 (C-10), 90.4 (C-12), 77.7 (C-11), 73.0 (C-4), 64.6 (C-13), 63.1 (C-2), 58.0 (C-5), 43.6 (C-6), 38.7 (C-3), 27.8 (C-8), 25.3 (C-7), 23.3 (C-16), 21.0 (C-2′), 13.6 (C-15), 10.8 (C-14); HRMS(ESI^+^): *m/z* 311.1837 [M + H]^+^ (calcd for C_17_H_27_O_5_, 311.1858).

#### 3.4.3. General Procedure for the Preparation of Compounds **4**–**5** [21]

On a stirring solution of trichodermin (**1**) (74 mg, 0.25 mmol) in dry chloroform (CHCl_3_, 6 mL), m-chloroperbenzoic acid (mCPBA) (123 mg, 70%, 0.5 mmol) was added at room temperature, and the reaction was monitored by TLC. After 6 h, 2 mL of a saturated solution of sodium hydrogen carbonate (NaHCO_3_) was added to the reaction mixture and was then followed by the addition of sodium sulphite (NaSO_3_). Distilled water (5 mL) was added, and the aqueous phase was extracted with CHCl_3_. The organic layer was dried over anhydrous Na_2_SO_4_, filtered, and the solvent was evaporated under reduced pressure. Column chromatography of the crude reaction mixture on silica gel, eluted with a gradient mixture of petroleum ether–EtOAc, produced a mixture of compounds **4** and **5**. Further purification with normal phase HPLC with petroleum ether/EtOAc (80:20) as mobile phase yielded pure compounds **4** and **5**.

*(4R,9S,10R)-9,10-Epoxy-12,13-epoxytrichothec-9-en-4-yl acetate* (**4**). (5.6 mg, 7.2%) Colourless oil; [α]_D_^20^ − 12.9° (*c* 0.09, CHCl_3_); IR (film) *υ*_max_ 2959, 1736, 1244, 1219, 1089 cm^−1^; ^1^H-NMR (CDCl_3,_ 400 MHz) δ 5.55 (1H, dd, *J* = 3.6, 7.8 Hz, H-4), 3.84 (1H, d, *J* = 5.1 Hz, H-2), 3.78 (1H, br. s, H-11), 3.11 (1H, d, *J* = 4.0 Hz, H-13), 2.93 (1H, d, *J* = 1.8 Hz, H-10), 2.79 (1H, d, *J* = 4.0 Hz, H-13), 2.50 (1H, dd, *J* = 7.8, 15.5 Hz, H-3), 2.05 (3H, s, H-2′), 2.00 (2H, m, H-3, H-7), 1.76 (2H, m, H-7,H-8), 1.33 (3H, s, H-16), 1.20 (1H, m, H-8), 1.05 (3H, s, H-15), 0.64 (3H, s, H-14); ^13^C-NMR (CDCl_3_, 100 MHz) δ 170.8 (C-1′), 79.2 (C-2), 74.7 (C-4), 70.2 (C-11), 65.0 (C-12), 61.5 (C-10), 57.3 (C-9), 49.5 (C-5), 47.8 (C-13), 40.3 (C-6), 36.3 (C-3), 25.8 (C-7), 24.2 (C-16), 24.2 (C-8), 21.1 (C-2′), 16.2 (C-15), 5.5 (C-14); HRMS (APGC^+^): *m/z* 309.1706 [M + H]^+^ (calcd for C_17_H_25_O_5_, 309.1702); 

*(4R,9R,10S)-9,10-Epoxy-12,13-epoxytrichothec-9-en-4-yl acetate* (**5**). (63.4 mg, 82.3%). Colourless oil; [α]_D_^20^ − 1.5° (*c* 0.65, CHCl_3_); IR (film) *υ*_max_ 2971, 1732, 1245, 1220, 1093 cm^−1^; ^1^H-NMR (CDCl_3,_ 400 MHz) δ 5.55 (1H, dd, *J* = 3.7, 7.9 Hz, H-4), 3.89 (1H, d, *J* = 5.2 Hz, H-2), 3.60 (1H, dd, *J* = 2.2, 5.6 Hz, H-11), 3.15 (1H, d, *J* = 4.0 Hz, H-13), 3.05 (1H, d, *J* = 5.6 Hz, H-10), 2.75 (1H, d, *J* = 4.0 Hz, H-13), 2.48 (1H, dd, *J* = 7.9, 15.4 Hz, H-3), 2.06 (3H, s, H-2′), 1.98 (1H, m, H-3), 1.88 (1H, m, H-7), 1.70 (2H, m, H-7,H-8), 1.33 (3H, s, H-16), 1.09 (1H, m, H-8), 0.88 (3H, s, H-15), 0.64 (3H, s, H-14); ^13^C NMR (CDCl_3_, 100 MHz) δ 170.9 (C-1′), 78.8 (C-2), 74.8 (C-4), 70.3 (C-11), 64.9 (C-12), 57.2 (C-10), 57.2 (C-9), 48.6 (C-5), 47.7 (C-13), 40.1 (C-6), 36.4 (C-3), 26.5 (C-7), 22.4 (C-16), 21.4 (C-8), 21.1 (C-2′), 16.8 (C-15), 5.7 (C-14); HRMS (APGC^+^): *m/z* 309.1706 [M+H]^+^ (calcd for C_17_H_25_O_5_, 309.1702).

#### 3.4.4. Preparation of Trichodermol (**2**)

A methanolic solution of sodium hydroxide (NaOH) (2 M, 6 mL) was added to trichodermin (**1**) (50 mg, 0.171 mmol), and the mixture was stirred at room temperature. After 45 min, an aqueous solution of hydrochloric acid (HCl) 1 N was added dropwise until reaching pH = 7. The methanol was evaporated under reduced pressure, and the aqueous suspension was extracted with EtOAc. The organic layer was dried over anhydrous Na_2_SO_4_, filtered, and the solvent was evaporated under reduced pressure. Column chromatography of the crude reaction mixture on silica gel, eluted with a mixture of petroleum ether–EtOAc (50:50), yielded white crystals (42.5 mg, 99%). Spectroscopic methods, specifically ^1^H-NMR and ^13^C-NMR, were then employed to identify the molecule. Spectroscopic data for the compound were identical to those described in the literature for trichodermol **2** [27]. 

#### 3.4.5. Preparation of Compound **6**

On a stirring solution of trichodermol (2) (20 mg, 0.08 mmol) in dry dichloromethane (CH_2_Cl_2_, 1 mL), *m*-chloroperbenzoic acid (mCPBA) (39.4 mg, 70%, 0.16 mmol) was added at room temperature, and the reaction was monitored by TLC. After 6 h, 1 mL of a saturated solution of sodium hydrogen carbonate (NaHCO_3_) was added to the reaction mixture, followed by sodium sulfite (NaSO_3_). Distilled water (5 mL) was added, and the aqueous phase was extracted with CH_2_Cl_2_. The organic layer was dried over anhydrous Na_2_SO_4_, filtered, and the solvent was evaporated under reduced pressure. Column chromatography of the crude reaction mixture on silica gel, eluted with a gradient mixture of petroleum ether–EtOAc, yielded pure compound **6**. 

*(4R,9R,10S)-9,10-Epoxy-12,13-epoxytrichothec-9-en-4-ol* (**6**). (63.4 mg, 82.3%). White solid; [α]_D_^20^ − 17.8° (*c* 0.17, CHCl_3_); IR (film) *υ*_max_ 3508, 2964, 2935, 1086, 746 cm^−1^; ^1^H-NMR (CDCl_3,_ 400 MHz) δ 4.29 (1H, dd, *J* = 3.2, 7.6 Hz, H-4), 3.90 (1H, d, *J* = 5.2 Hz, H-2), 3.51 (1H, dd, *J* = 2.3, 5.6 Hz, H-11), 3.13 (1H, d, *J =* 3.8 Hz, H-13), 3.03 (1H, d, *J* = 5.6 Hz, H-10), 2.74 (1H, d, *J =* 3.8 Hz, H-13), 2.56 (1H, dd, *J* = 7.6, 15.8 Hz, H-3), 1.90 (2H, m, H-3, H-7), 1.71 (2H, m, H-7,H-8), 1.32 (3H, s, H-16), 1.12 (1H, m, H-8), 0.81 (3H, s, H-15), 0.73 (3H, s, H-14).; ^13^C-NMR (CDCl_3_, 100 MHz) δ 78.38 (C-2), 73.98 (C-4), 70.05 (C-11), 65.17 (C-12), 57.25 (C-10), 57.18 (C-9), 48.82 (C-5), 47.42 (C-13), 39.96 (C-3), 39.49 (C-6), 26.60 (C-7), 22.43 (C-16), 21.35 (C-8), 16.62 (C-15), 6.23 (C-14); HRMS (ESI^+^): *m/z* 267.1591 [M + H]^+^ (calcd for C_15_H_23_O_4_, 267.1596).

#### 3.4.6. General Procedure for the Preparation of Trichodermol (**2**) C-4 derivatives (**7**–**15**)

A solution of trichodermol (**2**) in dry CH_2_Cl_2_ was prepared in a Schlenk flask under an inert argon atmosphere. Dry pyridine (d = 0.982 g/mL) was added, followed by the dropwise addition of acyl halide. The suspension was stirred at room temperature and monitored by TLC. When the reaction was completed, the crude mixture was supported on silica gel, and the products were separated by column chromatography and eluted with a gradient mixture of petroleum ether–EtOAc. Each product (**7**–**15**) was purified by HPLC. See supporting information, S2, for more details and yields.

*(4R)-12,13-Epoxytrichothec-9-en-4-ylpropionate* (**7**). Colourless oil; [α]_D_^20^ − 7.6° (*c* 0.25, CHCl_3_); IR (film) *υ*_max_ 3392, 2979, 1731, 1192 cm^−1^; ^1^H-NMR (CDCl_3_, 400 MHz) δ 5.56 (1H, dd, *J* = 3.8, 7.9 Hz, H-4), 5.39 (1H, m, H-10), 3.80 (1H, d, *J* = 5.3 Hz, H-2), 3.59 (1H, d, *J* = 5.6 Hz, H-11), 3.10 (1H, d, *J* = 4.1 Hz, H-13), 2.81 (1H, d, *J* = 4.1 Hz, H-13), 2.52 (1H, dd, *J* = 7.9, 15.5 Hz, H-3), 2.35 (2H, q, *J* = 7.6 Hz, H-2′), 1.94 (4H, m, H-3, H-7, H-8), 1.70 (3H, s, H-16), 1.39 (1H, m, H-7), 1.14 (3H, t, *J* = 7.6 Hz, H-3′), 0.92 (3H, s, H-15), 0.69 (3H, s, H-14).; ^13^C-NMR (CDCl_3_, 100 MHz) δ 174.3 (C-1′), 140.2 (C-9), 118.6 (C-10), 79.1 (C-2), 74.8 (C-4), 70.5 (C-11), 65.5 (C-12), 48.9 (C-5), 47.8 (C-13), 40.4 (C-6), 36.7 (C-3), 27.9 (C-8), 27.7 (C-2), 24.4 (C-7), 23.2 (C-16), 15.9 (C-15), 9.1 (C-3′), 5.8 (C-14); HRMS (APGC^+^): *m/z* 307.1917 [M + H]^+^ (calcd for C_18_H_27_O_4_, 307.1909).

*(4R)-12,13-Epoxytrichothec-9-en-4-ylbutyrate* (**8**). Colourless oil; [α]_D_^20^ − 4.6° (*c* 0.06, CHCl_3_); IR (film) *υ*_max_ 2961, 1729, 1176, 1078 cm^−1^; ^1^H-NMR (CDCl_3_, 400 MHz) δ 5.56 (1H, dd, *J* = 4.0, 8.0 Hz, H-4), 5.39 (1H, m, H-10), 3.81 (1H, d, *J* = 5.2 Hz, H-2), 3.60 (1H, d, *J* = 5.6 Hz, H-11), 3.11 (1H, d, *J* = 4.0 Hz, H-13), 2.81 (1H, d, *J* = 4.0 Hz, H-13), 2.52 (1H, dd, *J* = 7.6, 15.2 Hz, H-3), 2.31 (2H, t, *J* = 7.2 Hz, H-2′), 1.92 (4H, m, H-3, H-7, H-8), 1.70 (3H, s, H-16), 1.65 (2H, td, *J* = 7.6 Hz, H-3′), 1.40 (1H, m, H-7), 0.94 (3H, t, *J* = 7.2 Hz, H-4′), 0.92 (3H, s, H-15), 0.69 (3H, s, H-14).; ^13^C-NMR (CDCl_3_, 100 MHz) δ 173.5 (C-1′), 140.2 (C-9), 118.6 (C-10), 79.1 (C-2), 74.8 (C-4), 70.5 (C-11), 65.5 (C-12), 48.9 (C-5), 47.8 (C-13), 40.4 (C-6), 36.7 (C-3), 36.3 (C-2′), 28.0 (C-8), 24.4 (C-7), 23.2 (C-16), 18.4 (C-3′), 16.0 (C-15), 13.6 (C-4′), 5.8 (C-14); HRMS (APGC^+^): *m/z* 321.2077 [M + H]^+^ (calcd for C_19_H_29_O_4_, 321.2066).

*(4R)-12,13-Epoxytrichothec-9-en-4-yl pentanoate* (**9**) [21]. Colourless oil; [α]_D_^20^ − 12.5° (*c* 0.22, CHCl_3_); IR (film) *υ*_max_ 2959, 1731, 1172, 1079 cm^−1^; ^1^H-NMR (CDCl_3_, 400 MHz) δ 5.55 (1H, dd, *J* = 3.6, 7.8 Hz, H-4), 5.38 (1H, m, H-10), 3.80 (1H, d, *J* = 5.2 Hz, H-2), 3.59 (1H, d, *J* = 5.2 Hz, H-11), 3.10 (1H, d, *J* = 4.0 Hz, H-13), 2.80 (1H, d, *J* = 4.0 Hz, H-13), 2.51 (1H, dd, *J* = 7.8, 15.4 Hz, H-3), 2.32 (2H, td, *J* = 7.4 Hz, H-2′), 1.90 (4H, m, H-3, H-7, H-8), 1.69 (3H, s, H-16), 1.59 (2H, m, *J* = 7.6 Hz, H-3′), 1.31 (3H, m, H-7, H-4′), 0.92 (3H, s, H-15), 0.89 (3H, t, *J* = 7.4 Hz, H-5′), 0.69 (3H, s, H-14); ^13^C-NMR (CDCl_3_, 100 MHz) δ 173.7 (C-1′), 140.1 (C-9), 118.6 (C-10), 79.1 (C-2), 74.8 (C-4), 70.4 (C-11), 65.5 (C-12), 48.9 (C-5), 47.8 (C-13), 40.4 (C-6), 36.7 (C-3), 34.1 (C-2′), 27.9 (C-8), 26.9 (C-3′), 24.4 (C-7), 23.2 (C-16), 22.2 (C-4′), 15.9 (C-15), 13.6 (C-5′), 5.8 (C-14); HRMS (APGC^+^): *m/z* 335.2237 [M + H]^+^ (calcd for C_20_H_31_O_4_, 335.2222).

*(4R)-12,13-Epoxytrichothec-9-en-4-yl hexanoate* (**10**). Colourless oil; [α]_D_^20^ − 14.5° (*c* 0.25, CHCl_3_); IR (film) *υ*_max_ 2957, 1732, 1171, 1080 cm^−1^; ^1^H-NMR (CDCl_3_, 400 MHz) δ 5.55 (1H, dd, *J* = 3.6, 7.8 Hz, H-4), 5.38 (1H, m, H-10), 3.80 (1H, d, *J* = 5.2 Hz, H-2), 3.58 (1H, d, *J* = 5.5 Hz, H-11), 3.10 (1H, d, *J* = 4.0 Hz, H-13), 2.80 (1H, d, *J* = 4.0 Hz, H-13), 2.51 (1H, dd, *J* = 7.8, 15.4 Hz, H-3), 2.31 (2H, td, *J* = 7.4 Hz, H-2′), 1.92 (4H, m, H-3, H-7, H-8), 1.69 (3H, s, H-16), 1.61 (2H, m, H-3′), 1.38 (1H, m, H-7), 1.28 (4H, m, H-4′, H-5′), 0.92 (3H, s, H-15), 0.87 (3H, t, *J* = 7.2 Hz, H-6′), 0.69 (3H, s, H-14); ^13^C-NMR (CDCl_3_, 100 MHz) δ 173.7 (C-1′), 140.1 (C-9), 118.5 (C-10), 79.1 (C-2), 74.8 (C-4), 70.5 (C-11), 65.5 (C-12), 48.9 (C-5), 47.8 (C-13), 40.4 (C-6), 36.6 (C-3), 34.3 (C-2′), 31.2 (C-4′),27.9 (C-8), 24.5 (C-3′), 24.4 (C-7), 23.2 (C-16), 22.2 (C-5′), 15.9 (C-15), 13.8 (C-6′), 5.8 (C-14); HRMS (APGC^+^): *m/z* 349.2390 [M + H]^+^ (calcd for C_21_H_33_O_4_, 349.2379).

*(4R)-12,13-Epoxytrichothec-9-en-4-yl heptanoate* (**11**). Colourless oil; [α]_D_^20^ − 11.7° (*c* 0.25, CHCl_3_); IR (film) *υ*_max_ 2931, 1731, 1168, 1081 cm^−1^; ^1^H-NMR (CDCl_3_, 400 MHz) δ 5.55 (1H, dd, *J* = 3.8, 7.9 Hz, H-4), 5.38 (1H, m, H-10), 3.80 (1H, d, *J* = 5.3 Hz, H-2), 3.59 (1H, d, *J* = 5.3 Hz, H-11), 3.10 (1H, d, *J* = 4.1 Hz, H-13), 2.80 (1H, d, *J* = 4.1 Hz, H-13), 2.51 (1H, dd, *J* = 7.9, 15.5 Hz, H-3), 2.31 (2H, t, *J* = 7.3 Hz, H-2′), 1.95 (4H, m, H-3, H-7, H-8), 1.69 (3H, s, H-16), 1.60 (2H, m, H-3′), 1.39 (1H, m, H-7), 1.27 (6H, m, H-4′, H-5′, H-6′), 0.92 (3H, s, H-15), 0.86 (3H, m, H-7′), 0.69 (3H, s, H-14).; ^13^C-NMR (CDCl_3_, 100 MHz) δ 173.8 (C-1′), 140.2 (C-9), 118.5 (C-10), 79.1 (C-2), 74.8 (C-4), 70.5 (C-11), 65.5 (C-12), 48.9 (C-5), 47.8 (C-13), 40.4 (C-6), 36.7 (C-3), 34.4 (C-2′), 31.4 (C-5′), 28.7 (4′), 27.9 (C-8), 24.8 (C-3′), 24.4 (C-7), 23.2 (C-16), 22.4 (C-6′), 15.9 (C-15), 13.9 (C-7′), 5.8 (C-14); HRMS (APGC^+^): *m/z* 363.2537 [M + H]^+^ (calcd for C_22_H_35_O_4_, 363.2535).

*(4R)-12,13-Epoxytrichothec-9-en-4-yl acrylate* (**12**) [21]. Colourless oil; [α]_D_^20^ − 14.9° (*c* 0.13, CHCl_3_); IR (film) *υ*_max_ 2972, 1717, 1192, 1079 cm^−1^; ^1^H-NMR (CDCl_3_, 400 MHz) δ 6.42 (1H, dd, *J* = 1.5, 17.3 Hz, H-3′), 6.14 (1H, dd, *J* = 10.4, 17.3 Hz, H-2′), 5.83 (1H, dd, *J* = 1.5, 10.4 Hz, H-3′), 5.63 (1H, dd, *J* = 3.5, 7.9 Hz, H-4), 5.40 (1H, m, H-10), 3.83 (1H, d, *J* = 5.3 Hz, H-2), 3.62 (1H, d, *J* = 5.6 Hz, H-11), 3.12 (1H, d, *J* = 4.1 Hz, H-13), 2.82 (1H, d, *J* = 4.1 Hz, H-13), 2.56 (1H, dd, *J* = 7.9, 15.5 Hz, H-3), 1.98 (4H, m, H-3, H-7, H-8), 1.71 (3H, s, H-16), 1.41 (1H, m, H-7), 0.94 (3H, s, H-15), 0.72 (3H, s, H-14).; ^13^C-NMR (CDCl_3_, 100 MHz) δ 166.0 (C-1′), 140.2 (C-9), 131.0 (C-3′), 128.4 (C-2′), 118.6 (C-10), 79.1 (C-2), 75.3 (C-4), 70.5 (C-11), 65.5 (C-12), 49.2 (C-5), 47.8 (C-13), 40.4 (C-6), 36.6 (C-3), 28.0 (C-8), 24.4 (C-7), 23.2 (C-16), 16.0 (C-15), 5.8 (C-14); HRMS (APGC^+^): *m/z* 305.1748 [M + H]^+^ (calcd for C_18_H_25_O_4_, 305.1753).

*(4R)-12,13-Epoxytrichothec-9-en-4-yl (E)-but-2-enoate* (**13**) [21]. Colourless oil; [α]_D_^20^ − 17.8° (*c* 0.18, CHCl_3_); IR (film) *υ*_max_ 2968, 1716, 1180, 1079 cm^−1^; ^1^H-NMR (CDCl_3_, 400 MHz) δ 6.98 (1H, dq, *J* = 7.0, 15.4 Hz, H-3′), 5.86 (1H, dq, *J* = 1.8, 15.4 Hz, H-2′), 5.60 (1H, dd, *J* = 3.6, 8 Hz, H-4), 5.39 (1H, m, H-10), 3.81 (1H, d, *J* = 5.2 Hz, H-2), 3.61 (1H, d, *J* = 5.2 Hz, H-11), 3.11 (1H, d, *J* = 4.0 Hz, H-13), 2.81 (1H, d, *J* = 4.0 Hz, H-13), 2.54 (1H, dd, *J* = 7.6, 15.2 Hz, H-3), 1.97 (4H, m, H-3, H-7, H-8), 1.86 (3H, dd, *J* = 1.8, 7 Hz, H-4′), 1.70 (3H, s, H-16), 1.40 (1H, m, H-7), 0.93 (3H, s, H-15), 0.70 (3H, s, H-14); ^13^C-NMR (CDCl_3_, 100 MHz) δ 166.2 (C-1′), 145.1 (C-3′), 140.2 (C-9), 122.7 (C-2′), 118.6 (C-10), 79.1 (C-2), 74.7 (C-4), 70.5 (C-11), 65.5 (C-12), 49.1 (C-5), 47.8 (C-13), 40.4 (C-6), 36.7 (C-3), 28.0 (C-8), 24.4 (C-7), 23.2 (C-16), 17.9 (C-4′), 16.0 (C-15), 5.8 (C-14); HRMS (APGC^+^): *m/z* 319.1912 [M + H]^+^ (calcd for C_19_H_27_O_4_, 319.1909).

*(4R)-12,13-Epoxytrichothec-9-en-4-yl 3-methylbutanoate* (**14**). Colourless oil; [α]_D_^20^ − 16.8° (*c* 0.21, CHCl_3_); IR (film) *υ*_max_ 2960, 1731, 1189, 1080 cm^−1^; ^1^H-NMR (CDCl_3_, 400 MHz) δ 5.55 (1H, dd, *J* = 3.6, 7.8 Hz, H-4), 5.39 (1H, m, H-10), 3.80 (1H, d, *J* = 5.2 Hz, H-2), 3.59 (1H, d, *J* = 5.5 Hz, H-11), 3.10 (1H, d, *J* = 4.0 Hz, H-13), 2.81 (1H, d, *J* = 4.0 Hz, H-13), 2.53 (1H, dd, *J* = 7.9, 15.5 Hz, H-3), 2.21 (2H, d, *J* = 6.9 Hz, H-2′), 2.10 (1H, m, H-3′), 1.94 (4H, m, H-3, H-7, H-8), 1.70 (3H, s, H-16), 1.40 (1H, m, H-7), 0.95 (3H, s, H-4′*), 0.94 (3H, s, H-5′*), 0.92 (3H, s, H-15), 0.70 (3H, s, H-14); ^13^C-NMR (CDCl_3_, 100 MHz) δ 173.0 (C-1′), 140.1 (C-9), 118.6 (C-10), 79.1 (C-2), 74.8 (C-4), 70.5 (C-11), 65.5 (C-12), 48.8 (C-5), 47.8 (C-13), 43.4 (C-2′), 40.4 (C-6), 36.8 (C-3), 27.9 (C-8), 25.5 (C-3′), 24.4 (C-7), 23.2 (C-16), 22.4 (C-4′*), 22.3 (C-5′*), 15.9 (C-15), 5.9 (C-14); HRMS (APGC^+^): *m/z* 335.2222 [M + H]^+^ (calcd for C_20_H_28_O_4_, 335.2222).

*(4R)-12,13-Epoxytrichothec-9-en-4-yl 2-(benzyloxy)acetate* (**15**). Colourless oil; [α]_D_^20^ − 11.8° (*c* 0.27, CHCl_3_); IR (film) *υ*_max_ 3332, 2970, 1749, 1731, 1200, 1125, 1079 cm^−1^; ^1^H-NMR (CDCl_3_, 400 MHz) δ 7.36 (5H, m, H-5′, H-6′, H-7′, H-8′, H-9′), 5.67 (1H, dd, *J* = 3.6, 7.8 Hz, H-4), 5.40 (1H, m, H-10), 4.64 (2H, s, H-3′), 4.12 (2H, s, H-2′), 3.82 (1H, d, *J* = 5.2 Hz, H-2), 3.59 (1H, d, *J* = 5.6 Hz, H-11), 3.11 (1H, d, *J* = 4.0 Hz, H-13), 2.80 (1H, d, *J* = 4.0 Hz, H-13), 2.55 (1H, dd, *J* = 7.8, 15.4 Hz, H-3), 1.96 (4H, m, H-3, H-7, H-8), 1.70 (3H, s, H-16), 1.40 (1H, m, H-7), 0.94 (3H, s, H-15), 0.70 (3H, s, H-14); ^13^C-NMR (CDCl_3_, 100 MHz) δ 170.3 (C-1′), 140.2 (C-9), 137.0 (C-4′), 128.4 (C-5′, C-9′), 128.0 (C-6′, C8′), 127.9 (C-7′), 118.5 (C-10), 79.0 (C-2), 75.6 (C-4), 73.3 (C-3′), 70.5 (C-11), 67.0 (C-2′), 65.4 (C-12), 49.1 (C-5), 47.8 (C-13), 40.4 (C-6), 36.5 (C-3), 27.9 (C-8), 24.4 (C-7), 23.2 (C-16), 16.0 (C-15), 5.8 (C-14); HRMS (APGC^+^): *m/z* 399.2174 [M + H]^+^ (calcd for C_24_H_31_O_5_, 399.2171).

#### 3.4.7. Preparation of Compound **16**

A solution of trichodermol (**2**) (10 mg, 0.04 mmol) in dry CH_2_Cl_2_ (3 mL) was prepared in a Schlenk flask. Dry pyridine (100 µL, d = 0.982 g/mL) was added, followed by propanoyl chloride (100 µL, 98%, d = 1.059, 1.1 mmol). The suspension was stirred at room temperature overnight. The crude mixture was supported on silica gel, and the product was separated by column chromatography and eluted with a gradient mixture of petroleum ether–EtOAc. Further purification with normal phase HPLC with petroleum ether/EtOAc (80:20) as the mobile phase yielded pure compound **16**.

*(4R,12S)-12-Hydroxy-12-hydroxymethyltrichothec-9-en-4-yl propionate* (**16**) Colourless oil; [α]_D_^20^ + 5.9° (*c* 0.33, CHCl_3_); IR (film) *υ*_max_ 3479, 2969, 1732, 1183 cm^−1^; ^1^H-NMR (CDCl_3_, 400 MHz) δ 5.50 (1H, m, H-10), 5.13 (1H, dd, *J* = 4.0, 8.0 Hz, H-4), 4.15 (1H, dd, *J* = 6.0, 11.2 Hz, H-2), 3.87 (2H, m, H-13), 3.75 (1H, d, *J* = 5.2 Hz, H-11), 2.57 (1H, dt, *J* = 5.9, 11.9 Hz, H-3), 2.33 (2H, q, *J* = 10.4 Hz, H-2′), 2.14 (1H, t, OH-13), 1.93 (3H, m, H-3, H-8), 1.71 (1H, m, H-7), 1.71 (3H, s, H-16), 1.27 (1H, m, H-7), 1.16 (3H, s, H-14), 1.14 (3H, t, *J* = 7.6 Hz, H-3′), 0.75 (3H, s, H-15); ^13^C-NMR (CDCl_3_, 100 MHz) δ 173.5 (C-1′), 141.0 (C-9), 117.3 (C-10), 90.5 (C-12), 77.7 (C-11), 72.8 (C-4), 64.6 (C-13), 63.2 (C-2), 58.1 (C-5), 43.6 (C-6), 38.7 (C-3), 27.8 (C-8), 27.8 (C-2′), 25.3 (C-7), 23.3 (C-16), 13.7 (C-15), 10.8 (C-14), 9.1 (C-3′); HRMS (ESI^+^): *m/z* 347.1830 [M + Na]^+^ (calcd for C_18_H_28_O_5_Na, 347.1834).

### 3.5. Evaluation of Antimicrobial Activity

#### 3.5.1. Antifungal Assay

Sabouraud Dextrose Agar (SDA) plates were inoculated with *C. albicans* MY1055 and incubated for 24 h at 35 °C. The raised colonies were collected from the SDA plates and resuspended in modified RPMI-1640 medium, which was prepared with 20.8 g of RPMI medium (Sigma-Aldrich, Madrid, Spain), 13.4 g of YNB (Yeast Nitrogen Base), 80 mL of 1M HEPES, and 72 mL of 50% glucose. The volume was adjusted to 2 L with MilliQ water and filtered. The OD at 660 nm of the colony suspension was adjusted to 0.25 using RPMI-1640 modified medium as diluent and blank. This inoculum was then again diluted to 1:10 and kept on ice until it was used to inoculate the 96-well microtiter plates. For the assay, 90 μL/well of diluted 1:10 inoculum was mixed with 1.6 μL/well of compound solution in DMSO and 8.4 μL/well of modified RPMI-1640 medium. Amphotericin B and penicillin G were used as positive and negative internal controls, respectively. After distribution to the test plates of the inoculum, samples, and controls, the absorbance at T_0_ (zero time) was measured at a 612 nm DO with a Tecan Ultraevolution spectrophotometer. The plates were then incubated at 37 °C for 20 h. Following this incubation period, the plates were shaken on a Micromix-5 shaker (Marshall Scientific, Hampton, NH, USA), and the absorbance at T_f_ (final time) was again measured at the same OD as initially. Percent growth inhibition (PGI) was then calculated.

Compounds **1**–**16** were serially diluted in DMSO with a dilution factor of 2 to provide 10 concentrations starting at 64 μg/mL. MIC was defined as the lowest concentration of an antimicrobial or antifungal compound that inhibited ≥95% growth of a microorganism after overnight incubation. Data were analyzed using the Screener Genedata program (Genedata AG, Basel, Switzerland). In all the experiments performed in this work, the RZ ‘factor obtained was between 0.85 and 0.95.

#### 3.5.2. Antibacterial Test 

The bacterial strains (Methicillin-resistant *Staphylococcus aureus* MRSA MB5393, *Acinetobacter baumannii* MB5973, *Escherichia coli* MB2884 and MB5746, and *Pseudomonas aeruginosa* MB5919) were re-plated on Luria-Bertani agar plates (LBA, 40 g/L) and incubated at 37 °C overnight to obtain isolated colonies. The individual colonies of each microorganism were inoculated with 25 mL of Luria-Bertani medium (LB, 25 g/L) in 250 mL Erlenmeyer flasks and incubated overnight at 37 °C with shaking at 220 rpm. The bacterial inocula were then diluted to obtain the corresponding equivalents for the assay of approximately 1.1 × 10^6^ CFU/mL (MRSA) or 5–6 × 10^5^ CFU/mL (*A. baumannii*, *E. coli* and *P. aeruginosa*). For the assay, 90 μL/well of diluted inoculum were mixed with 1.6 μL/well of each compound dissolved in DMSO and 8.4 μL/well of LB medium. Kanamycin and amphotericin B (MRSA), rifampicin and amphotericin B (*A. baumannii*), novobiocin and amphotericin B (*E. coli*), and ciprofloxacin and amphotericin B (*P. aeruginosa*) were included as positive and negative internal controls respectively on each plate. The absorbance at T_0_ (zero time) was measured at 612 nm with a Tecan Ultra Evolution spectrophotometer and immediately thereafter, the plates were incubated at 37 °C for 20 h. After this period, the assay plates were shaken using a Micromix-5 agitator and once again the absorbance at T_f_ (final time) was measured at an OD of 612 nm. Percent growth inhibition was calculated and the MIC was defined in the same way as described for *C. albicans*. The data were also analyzed using the Screener Genedata program (Genedata AG, Switzerland). In all the experiments performed in this work, the RZ ‘factor obtained was between 0.85 and 0.95. Assays on agar plates were conducted to determine antimicrobial susceptibility against vancomycin resistant *B. subtilis* MB964 and β-lactam antibiotics. *B. subtilis* MB964 assay plates were prepared by adding 1 mL (1.5 × 10^8^ CFU/mL) of a suspension of *B. subtilis* spores directly to 1 L of yeast extract and nutrient agar medium (NAYE, nutrient agar 23 g/L, yeast extract 2 g/L) and pouring 30 mL of this mixture into each test plate. The compounds were serially diluted in 20% DMSO with a dilution factor of 2 to provide 10 compound concentrations starting at 64 μg/mL. Ten microliters of each compound concentration were added to the agar plate containing the pathogenic microorganism. Activity was determined by measuring the size of the inhibition halos.

#### 3.5.3. MTT Cytotoxicity Assay

The MTT tetrazole reduction rate is an indicator of the functional integrity of the mitochondria and, hence, of cellular viability. Day 1: Cells were seeded at their corresponding concentrations in 96-well plates and allowed to incubate for 24 h. Day 2: The media was stripped from the plates, and then the previously prepared compounds were added in 100% DMSO, serial ½ dilutions. Compounds were added in a 1:200 dilution (2 μL of compound/600 μL of medium) using a BiomekFX ™ multi-well automatic plate dispenser (Beckman Coulter). Of this mixture, 200 μL were pipetted to three-cell plates, resulting in each compound tested in triplicate with a constant DMSO percentage of 10.5%. We used MMS (2 mM) as a positive control and DMSO as negative control at the same concentration at which the compounds and controls had been dissolved. Day 3: Compounds were incubated with the cells for 24 h. The MTT was then added to a final concentration of 0.5 mg/mL and allowed to incubate with the cells for 3 h. The chromogenic formazan concentration generated by spectrophotometry at 570 nm was then determined in a Victor2 ™ multi-well plate reader (PerkinElmer). Data were analyzed using the ScreenerTM (Genedata) program for the calculation of IC_50_ (dose causing 50% cell death), taking as treatment 100% cell death (positive control) with 1 mM MMS, and as 0% cell death (negative control) 0.5% DMSO. The percentage of cell death for each concentration was calculated. Then, using the Levenberg-Marquardt algorithm to adjust the dose-response curve, the IC_50_ of each compound was calculated by interpolating the curve thus obtained.

## 4. Conclusions

The results presented in this paper point to trichodermin (**1**) as a molecule with great potential as a model in the development of new drugs. Our results show that, through simple modifications on the trichodermin skeleton (**1**), it is possible to modulate bioactivity and obtain derivatives that are of great interest in the research and development of new molecules with potential use as antimicrobial agents and/or cytotoxic cancer treatment drugs. The specificity observed in the biological activity of these trichothecenes should spark further study of their mechanisms of action through the use of other study models.

## Figures and Tables

**Table 1:** Antimicrobial activity of compounds **1, 2**, **7**–**9**, and **10**–**15**. The results are expressed as MIC in μg/mL.

Entry	*E. coli* MB5746	*MRSA* MB5393	*C. albicans* MY1055
**1**	>64	>64	<4
**2**	>64	>64	8–16
**7**	>64	>64	16–32
**9**	64	>64	4–8
**10**	32	>64	8
**11**	32	>64	>64
**12**	>64	>64	4
**13**	>64	>64	4
**14**	>64	>64	8–16
**15**	>64	>64	8

MRSA = Methicillin-resistant *S. aureus.* MIC = minimal inhibitory concentration.

**Table 2:** Cytotoxicity of compounds **1**, **2**, **7**, **9**–**15** in different human cell lines, measured at 24 h of incubation. IC_50_ (μg/mL).

Entry	HepG2	MCF-7	RCC4-VA	RCC4-VHL	Fa2N4
**1**	>20	<1.25	14.19	>20	12.09
**2**	>20	4.42	>20	>20	>20
**7**	>20	13.52	>20	>20	>20
**9**	>20	1.93	>20	>20	>20
**10**	14.20	3.59	>20	>20	>20
**11**	>20	11.26	>20	>20	>20
**12**	>20	3.78	6.02	8.00	>20
**13**	17.48	2.15	>20	>20	>20
**14**	>20	5.22	>20	>20	>20
**15**	>20	2.03	17.14	>20	>20

HepG2 = liver cancer (CCL-8065); MCF-7 = breast carcinoma (HTB-22); RCC4-VA and –VHL = renal carcinoma; Fa2N4 = immortalized human hepatocytes.

## Supplementary Materials

The following are available online at https://www.mdpi.com/1420-3049/24/20/3811/s1: Part A: General conditions for the preparation of compounds **7**–**15**. Part B: Antimicrobial and cytotoxic activities of compounds **1**–**16**. 1H and 13C-NMR spectra for compounds **3**–**16**.
