# Supplementary material for: Synthesis of Trichodermin Derivatives and Their Antimicrobial and Cytotoxic Activities
Source: Molecules. 2019 Oct 22;24(20):3811. doi: 10.3390/molecules24203811 (PMC6833013; doi:10.3390/molecules24203811)
Supplement: Supplementary file 1 [file molecules-24-03811-s001.pdf]

Synthesis of Trichodermin Derivatives and Their Antimicrobial and Cytotoxic Activities.

Javier E. Barúa [1,2] Mercedes de la Cruz [3], Nuria de Pedro [3], Bastien Cautain [3], Rosa Hermosa [4], Rosa E. Cardoza [5], Santiago Gutiérrez [5], Enrique Monte [4], Francisca Vicente [3], and Isidro G. Collado [2,*]

1 National University of Asunción, Biological Chemistry Department, Chemistry Science Faculty, Ruta Mcal. Estigarribia Km 11.5, San Lorenzo, 2160, Paraguay; javierbarua@qui.una.py
2 University of Cádiz, Campus of Puerto Real, Science Faculty, Department of Organic Chemistry, 11510, Puerto Real, Cádiz, Spain
3 Fundación Medina, Avda. del Conocimiento 34, 18016 Armilla, Granada, Spain; mercedes.delacruz@medinaandalucia.es (M.d.l.C.); nuri_dp@hotmail.es (N.d.P.); bastien.cautain@medinaandalucia.es (B.C.); francisca.vicente@medinaandalucia.es (F.V.)
4 Spanish-Portuguese Institute for Agricultural Research (CIALE), Department of Microbiology and Genetics, University of Salamanca, Campus of Villamayor, Rio Duero 12, 37185 Salamanca, Spain; rhp@usal.es (R.H.); emv@usal.es (E.M.)
5 University of León, Campus of Ponferrada, Superior and Technical Universitary School of Agricultural Engineers. Area of Microbiology, Avda. Astorga s/n, 24400 Ponferrada, Spain; re.cardoza@unileon.es (R.E.C.); s.gutierrez@unileon.es (S.G.)
* Correspondence: isidro.gonzalez@uca.es; Tel.: +34 956 012768

Table of contents:

Part A: General conditions for the preparation of compounds **7–15**……………….**S2**

Part B: Antimicrobial and cytotoxic activities of compounds **1–16**.....................**S3–S4**

Part C: ^{1}H and ^{13}C-NMR spectra for compounds **3–16**……………………......**S5–S18**

PART A : PREPARATION OF COMPOUNDS **7–15**

Table S1. Reaction condition data for preparation of **7**–**15.**

Entry	acyl halide (mmol)	Trichodermol (mmol)	Pyridine (μL)	CH_2Cl_2 (mL)	time (h)	yield (%)
**7**	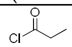 0.14	0.04	100	4	15	85
**8**	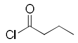 0.1	0.04	100	2	2	44.5
**9**	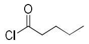 0.2	0.08	200	4	2	91.7
**10**	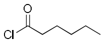 0.2	0.08	200	4	2	91.9
**11**	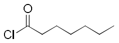 0.2	0.08	200	4	2	81.8
**12**	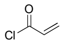 0.24	0.08	200	3	1	47.7
**13**	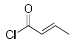 0.1	0.04	100	2	2	62
**14**	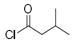 0.2	0.08	200	4	2	56.3
**15**	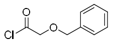 0.2	0.08	200	4	2	94

PART B: BIOLOGICAL ACTIVITIES

Table S2. Antimicrobial activity of compounds **1**-**16**. The results are expressed as MIC in μg/mL.

Entry	*A. baumannii* MB5973	*E.coli* MB2884	*E.coli* MB5746	*P. aeruginosa* MB5919	*S. aureus* EPI167	*MRSA* 5393	*C. albicans* MY1055	*B. subtilis* MB964
**1**	>64	>64	>64	>64	>64	>64	<4	no halo
**2**	>64	>64	>64	>64	>64	>64	8-16	no halo
**3**	>64	>64	>64	>64	>64	>64	>64	no halo
**4**	>64	>64	>64	>64	>64	>64	>64	no halo
**5**	>64	>64	>64	>64	>64	>64	>64	no halo
**6**	>64	>64	>64	>64	>64	>64	>64	no halo
**7**	>64	>64	>64	>64	>64	>64	16-32	no halo
**8**	>64	>64	nd	>64	>64	nd	nd	nd
**9**	>64	>64	64	>64	>64	>64	4–8	no halo
**10**	>64	>64	32	>64	>64	>64	8	no halo
**11**	>64	>64	32	>64	>64	>64	>64	no halo
**12**	>64	>64	>64	>64	>64	>64	4	no halo
**13**	>64	>64	>64	>64	>64	>64	4	no halo
**14**	>64	>64	>64	>64	>64	>64	8–16	no halo
**15**	>64	>64	>64	>64	>64	>64	8	no halo
**16**	>64	>64	>64	>64	>64	>64	>64	no halo

Nd = no data; MRSA= Methicillin-resistant *S. aureus;* MIC= minimal inhibitory concentration

Table S3. Cytotoxicity of compounds **1**-**16** in different human cell lines, measured at 24 hours of incubation. IC_{50} (µg/mL).

Entry	*A549*	*HepG2*	*HT29*	*MCF-7*	*RCC4-VA*	*RCC4-VHL*	*Fa2N4*
**1**	>20	>20	>20	<1.25	14.19	>20	12.09
**2**	>20	>20	>20	4.42	>20	>20	>20
**3**	>20	>20	>20	>20	>20	>20	>20
**5**	>20	>20	>20	>20	>20	>20	>20
**6**	>20	>20	>20	>20	>20	>20	>20
**7**	>20	>20	>20	13.52	>20	>20	>20
**9**	>20	>20	>20	1.93	>20	>20	>20
**10**	>20	14.20	>20	3.59	>20	>20	>20
**11**	>20	>20	>20	11.26	>20	>20	>20
**12**	>20	>20	>20	3.78	6.02	8.00	>20
**13**	>20	17.48	>20	2.15	>20	>20	>20
**14**	>20	>20	>20	5.22	>20	>20	>20
**15**	>20	>20	>20	2.03	17.14	>20	>20
**16**	>20	>20	>20	>20	>20	>20	>20

A549 = adenocarcinomic alveolar basal epithelial cells (CCL-185); HepG2= liver hepatocellular (CCL-8065); HT29= colorectal adenocarcinoma (HTB-38); MCF-7= breast carcinoma (HTB-22); RCC4-VA and RCC4-VHL = renal carcinoma; Fa2N4= immortalized human hepatocytes

PART C: ^{1}H AND ^{13}C-NMR SPECTRA

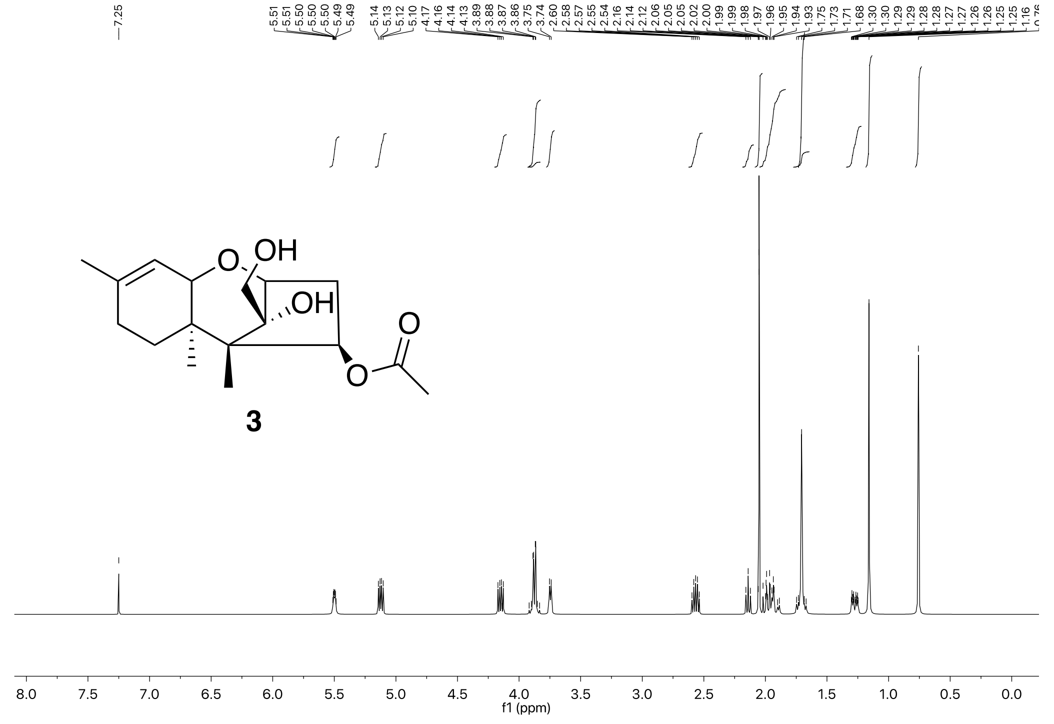

400 MHz ^{1}H NMR of compound **3** in $CDCl_3$

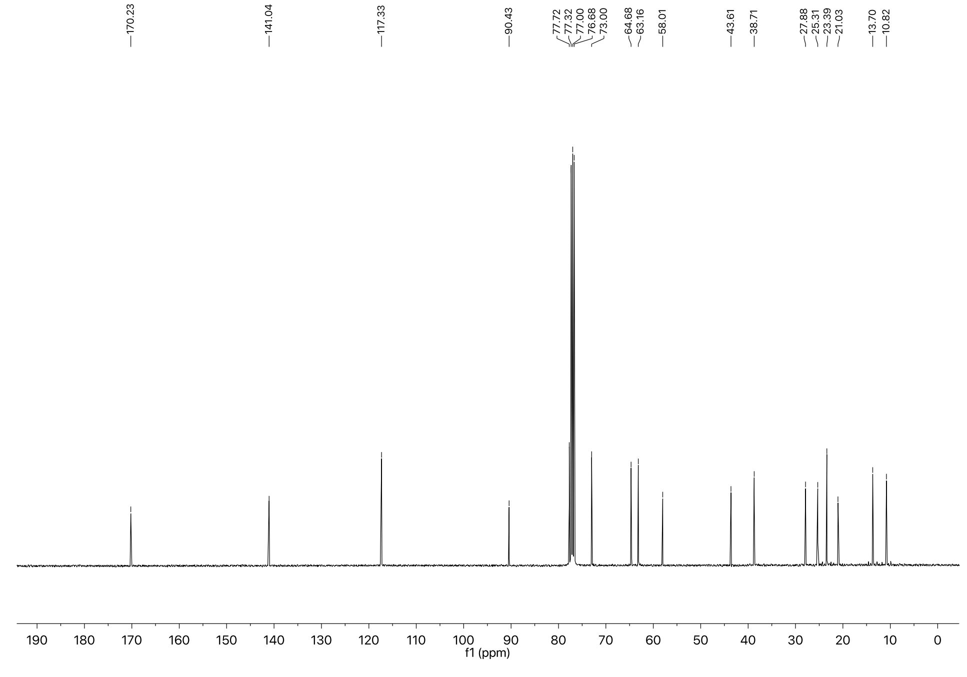

100 MHz ^{13}C NMR of compound **3** in $CDCl_3$

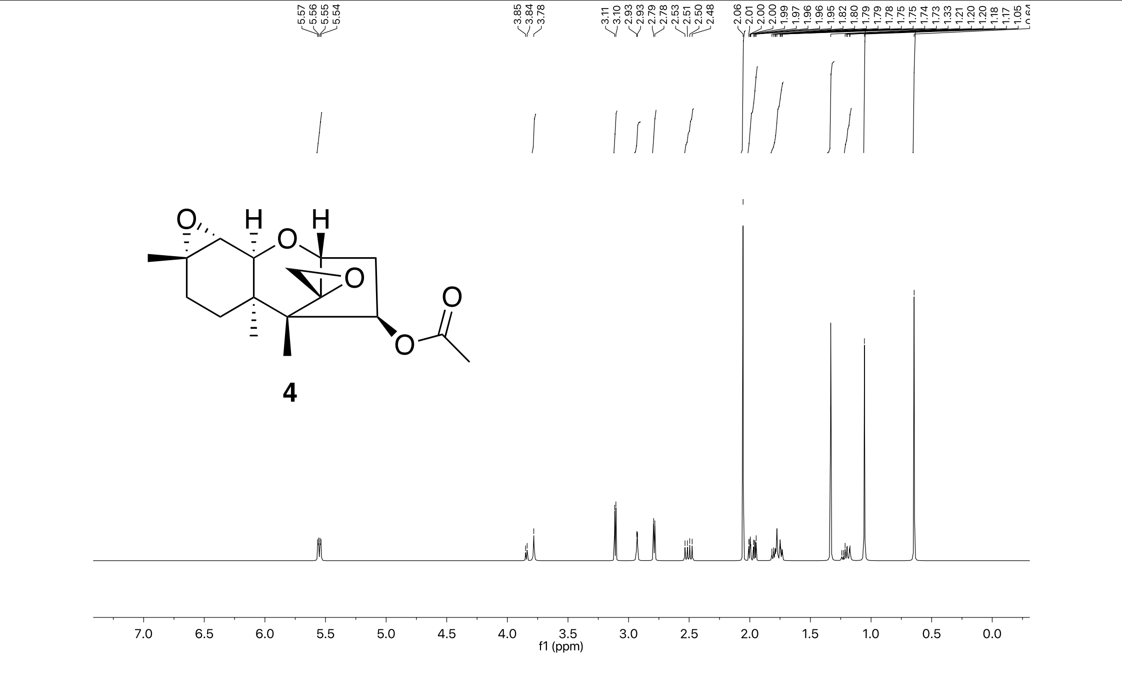

400 MHz ^{1}H NMR of compound **4** in $CDCl_3$

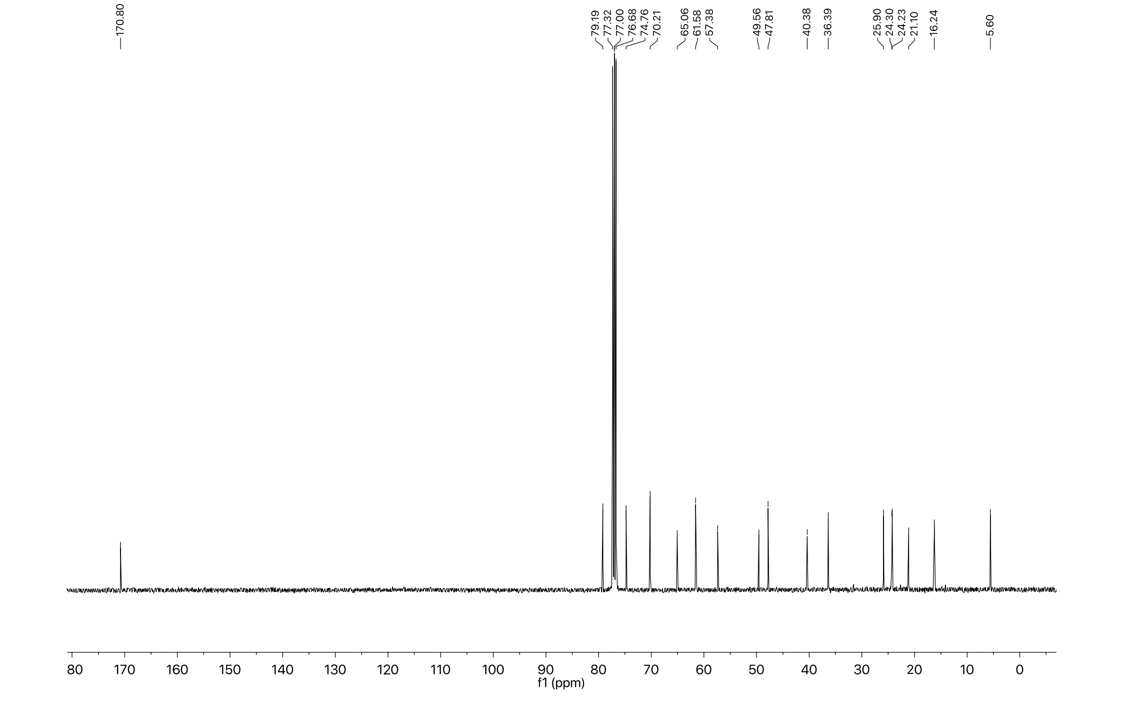

100 MHz ^{13}C NMR of compound **4** in $CDCl_3$

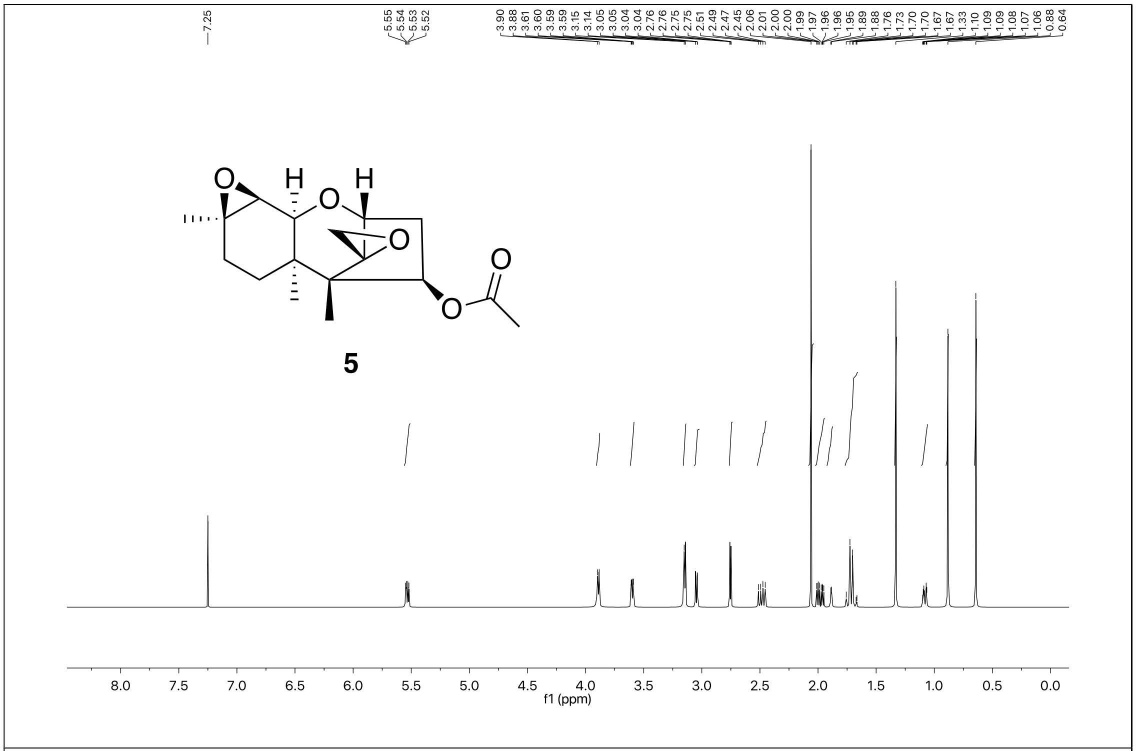

400 MHz ^{1}H NMR of compound **5** in $CDCl_3$

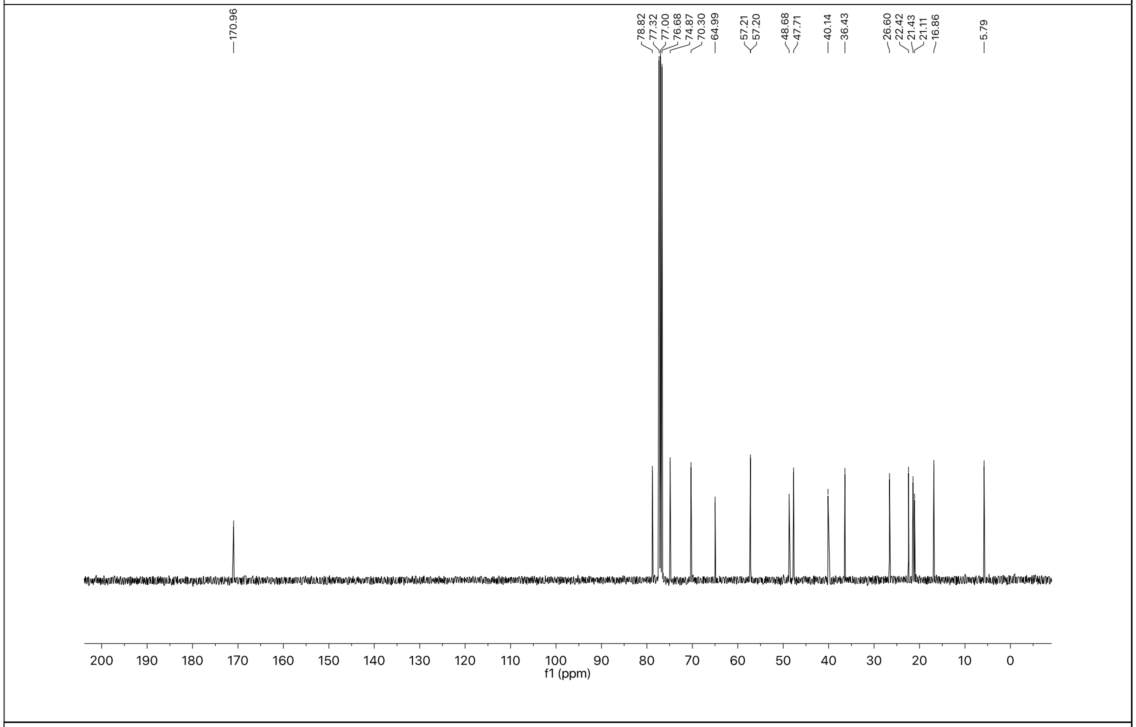

100 MHz ^{13}C NMR of compound **5** in $CDCl_3$

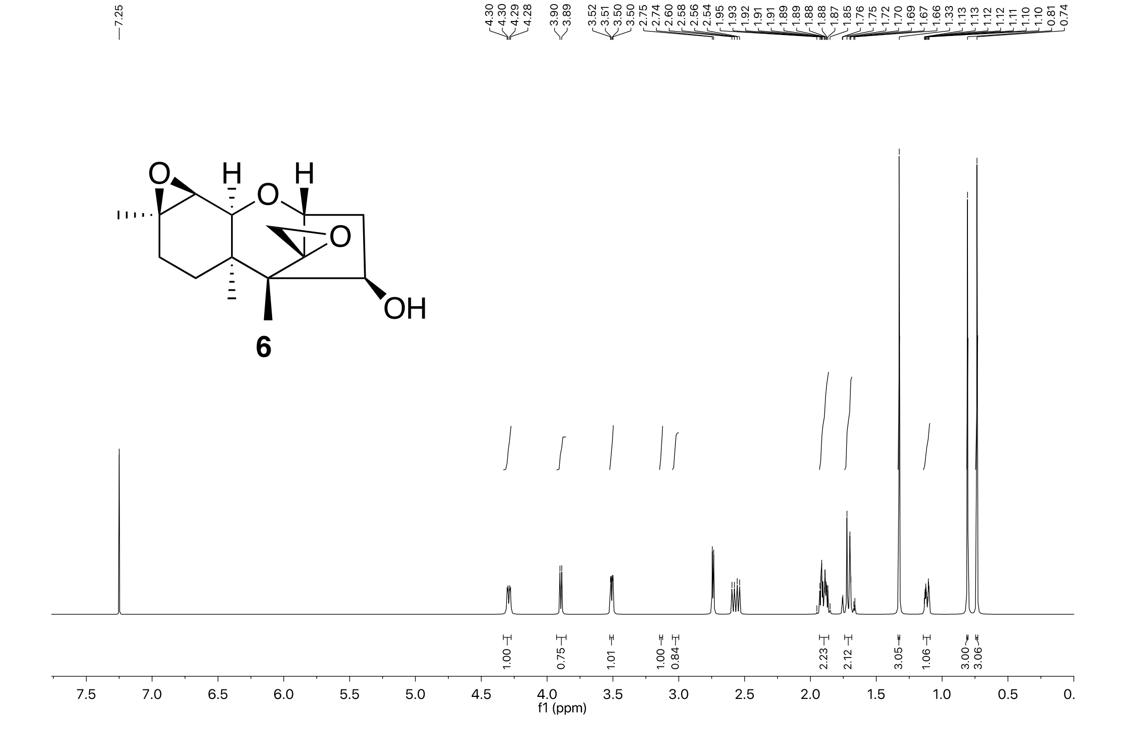

400 MHz ^{1}H NMR of compound **6** in $CDCl_3$

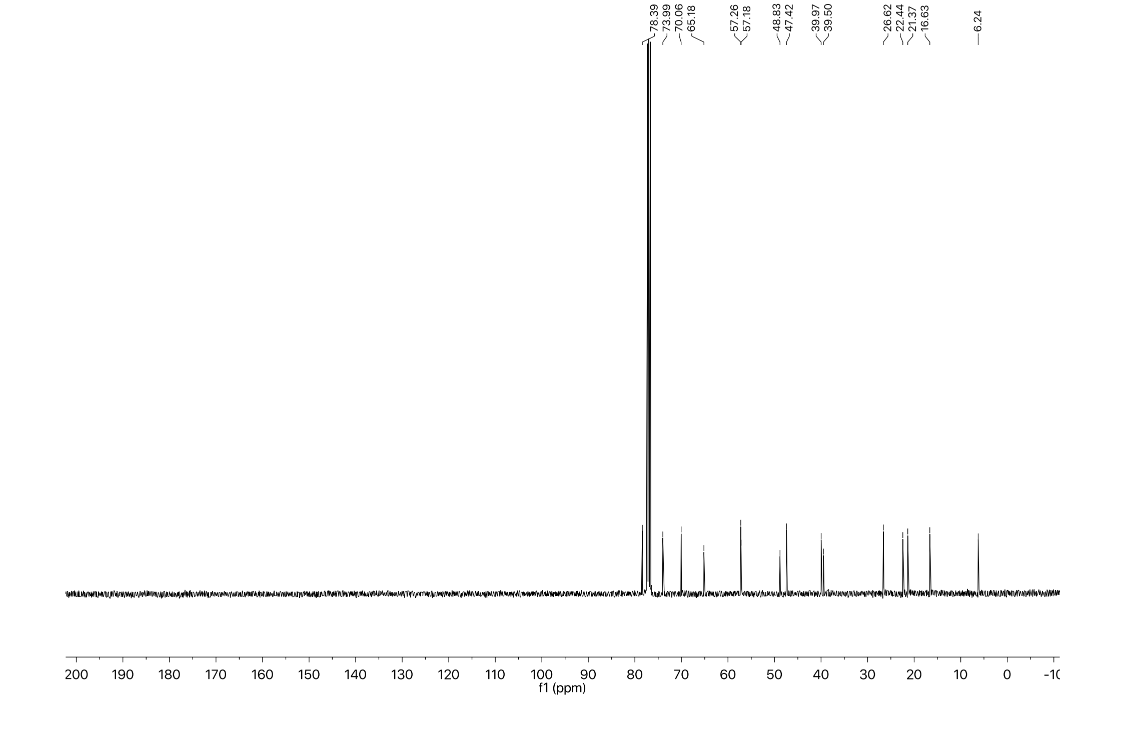

100 MHz ^{13}C NMR of compound **6** in $CDCl_3$

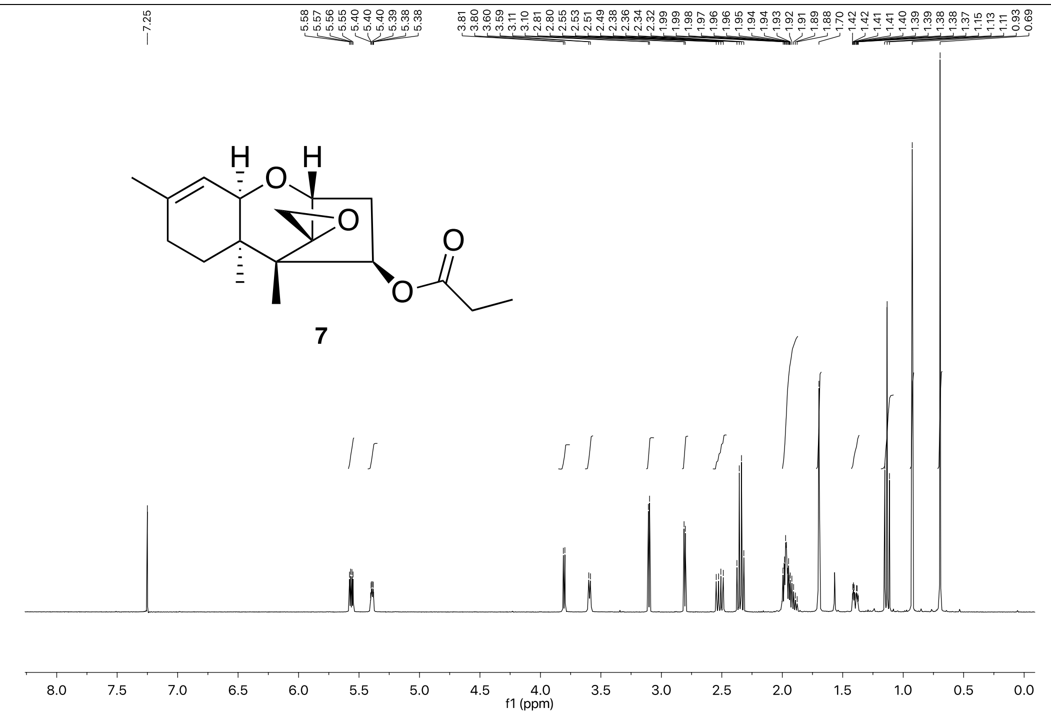

400 MHz ^{1}H NMR of compound **7** in $CDCl_3$

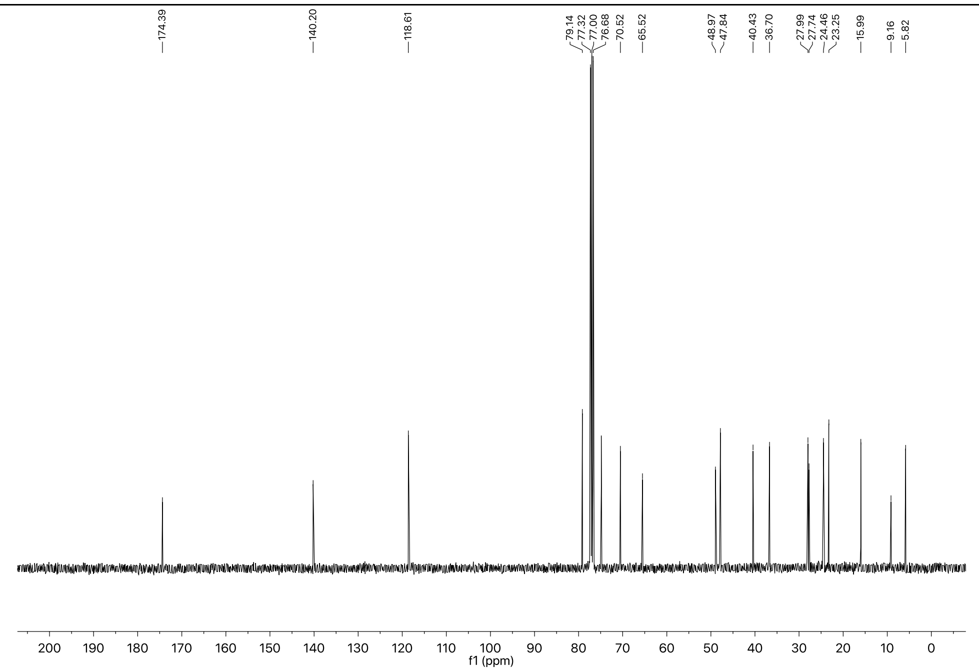

100 MHz ^{13}C NMR of compound **7** in $CDCl_3$

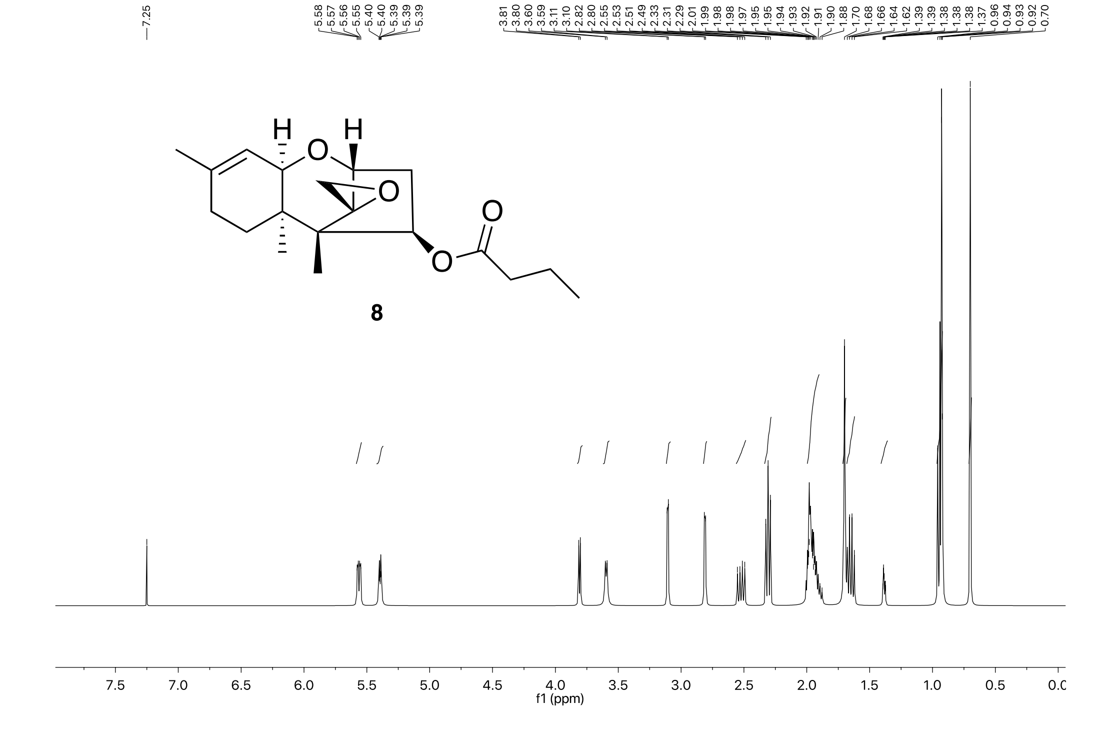

400 MHz ^{1}H NMR of compound **8** in $CDCl_3$

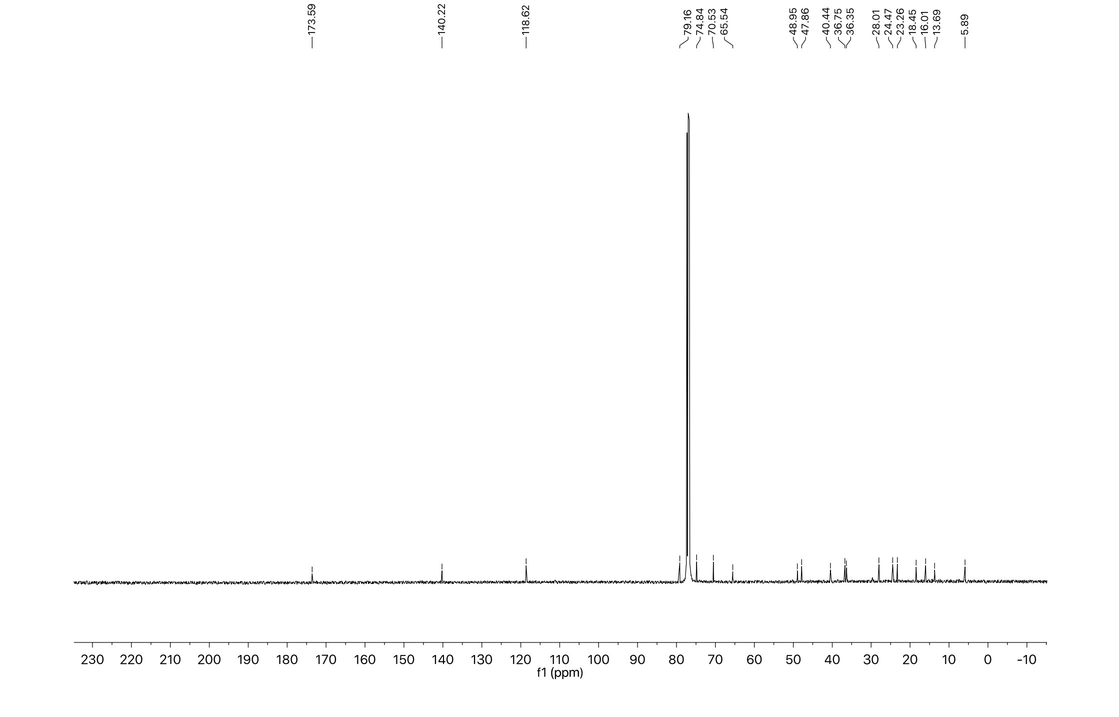

100 MHz ^{13}C NMR of compound **8** in $CDCl_3$

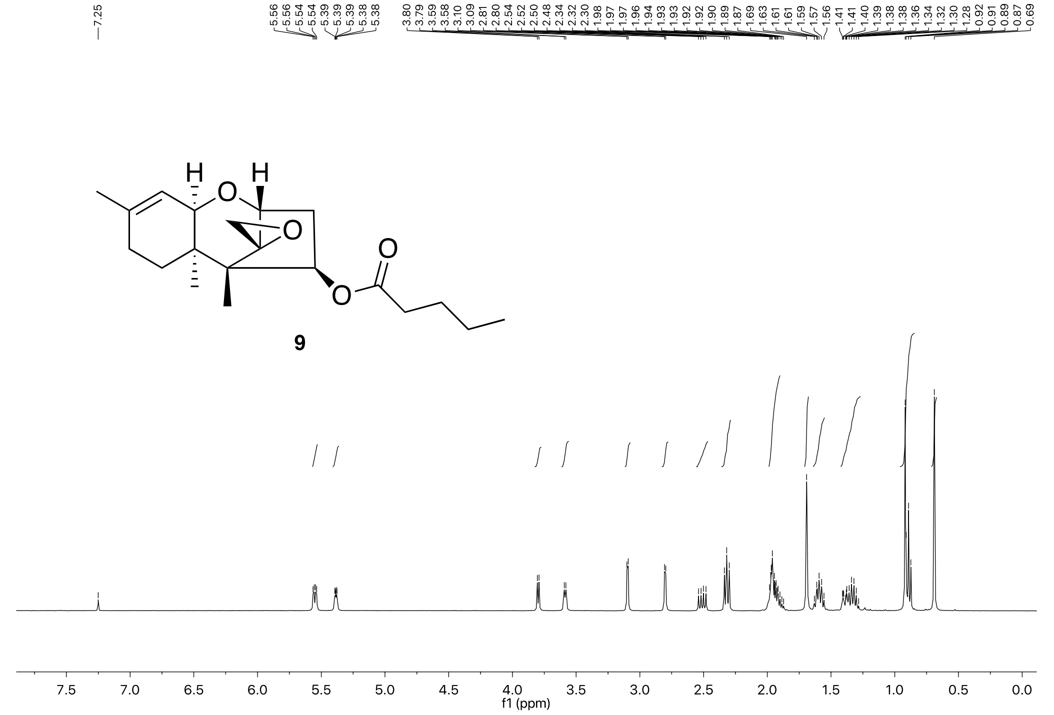

400 MHz ^{1}H NMR of compound **9** in $CDCl_3$

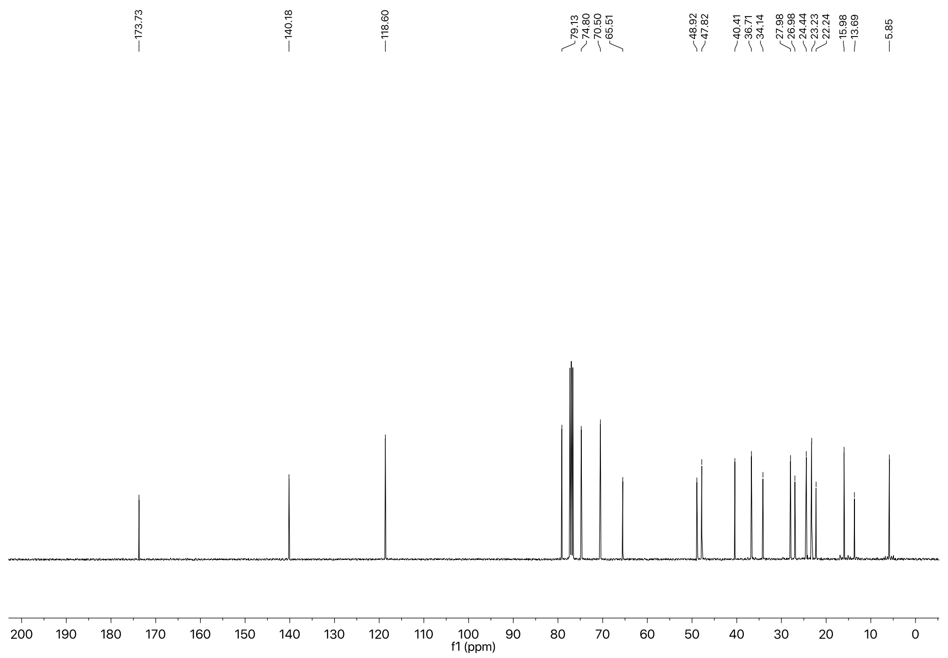

100 MHz ^{13}C NMR of compound **9** in $CDCl_3$

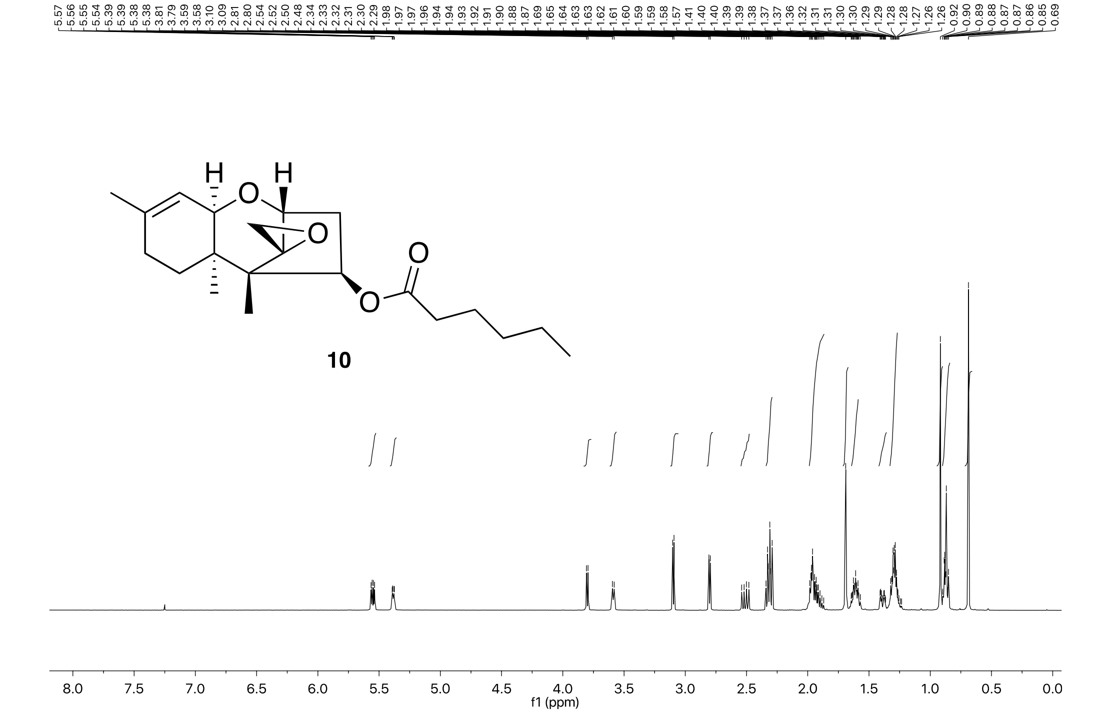

400 MHz ^{1}H NMR of compound **10** in $CDCl_3$

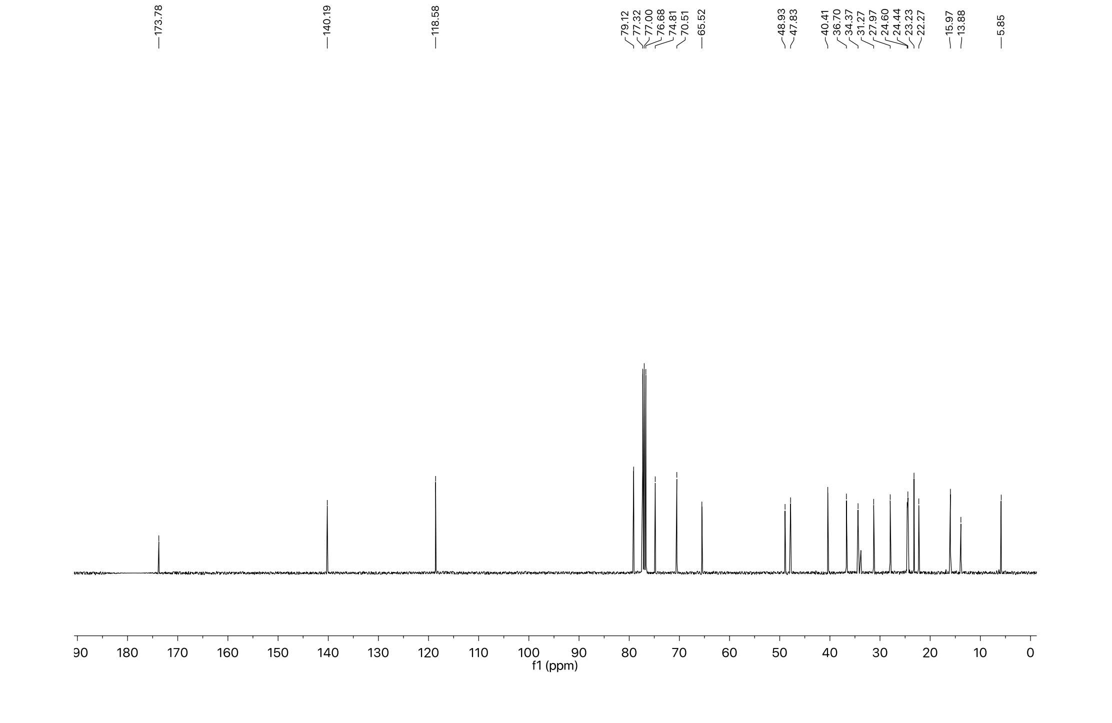

100 MHz ^{13}C NMR of compound **10** in $CDCl_3$

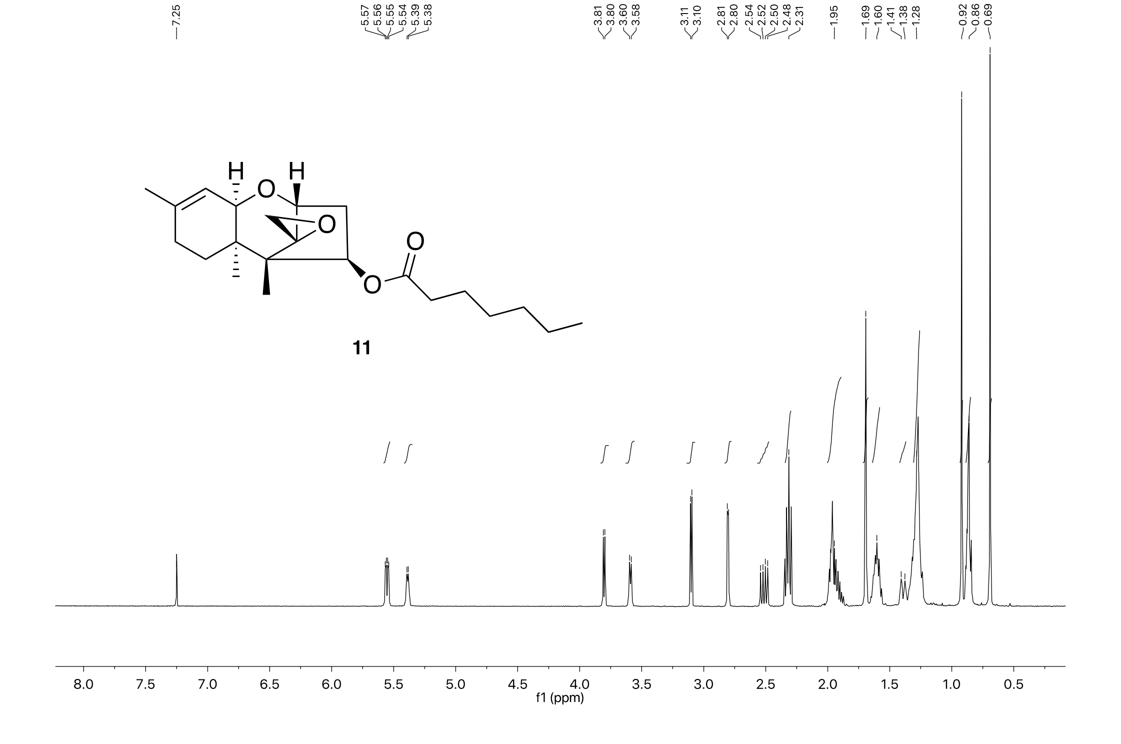

400 MHz ^{1}H NMR of compound **11** in $CDCl_3$

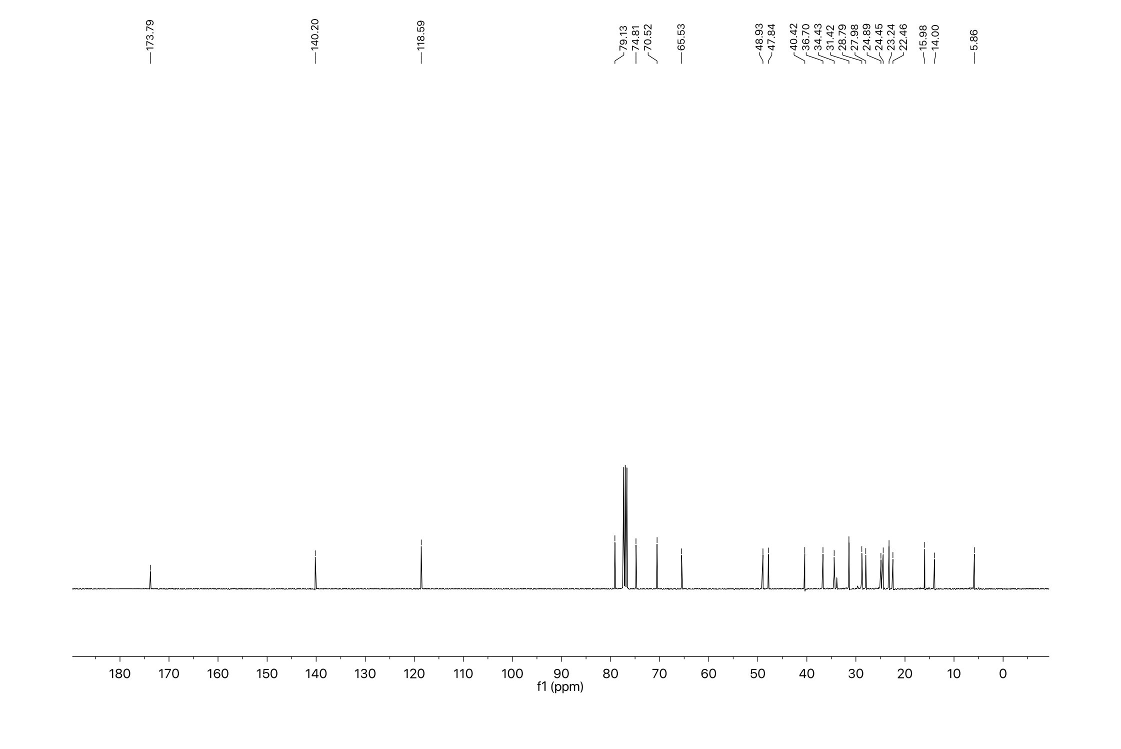

100 MHz ^{13}C NMR of compound **11** in $CDCl_3$

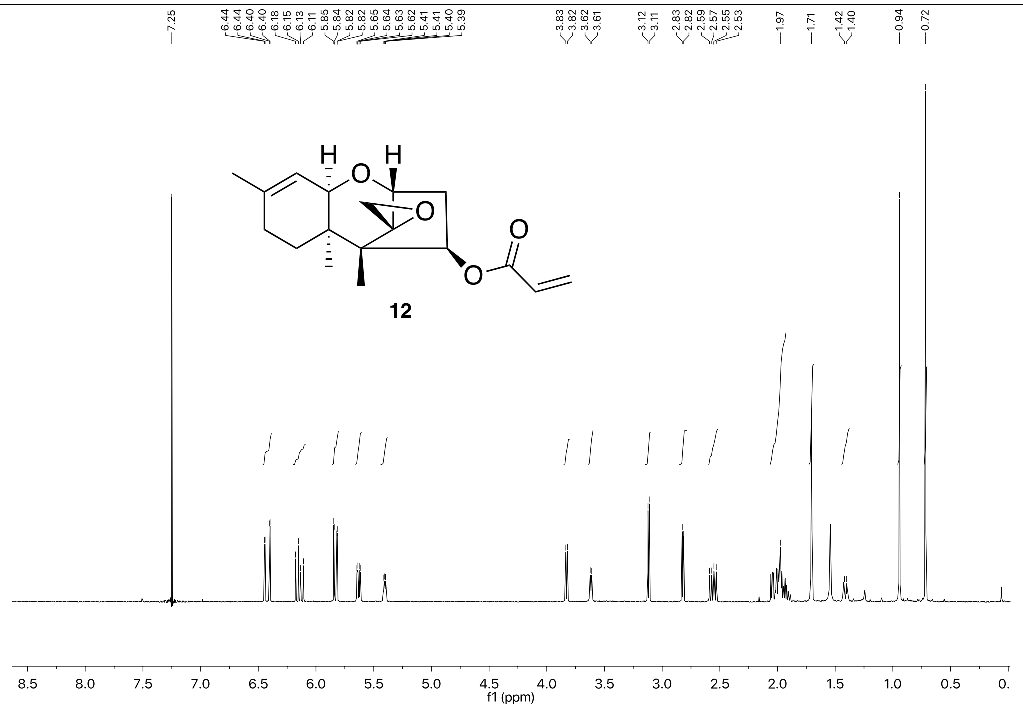

400 MHz ^{1}H NMR of compound **12** in $CDCl_3$

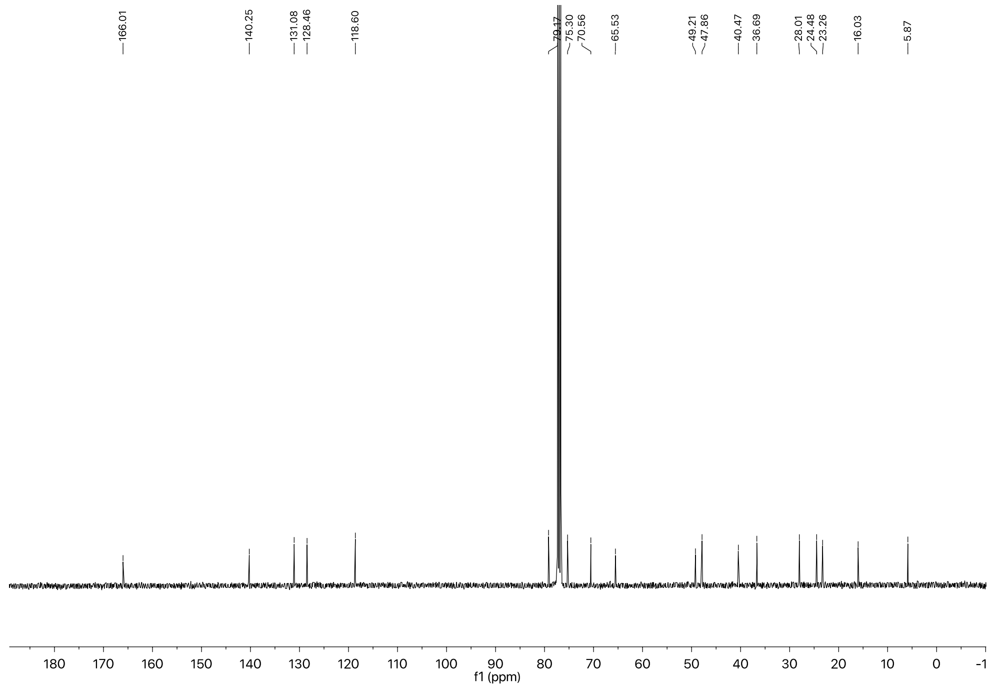

100 MHz ^{13}C NMR of compound **12** in $CDCl_3$

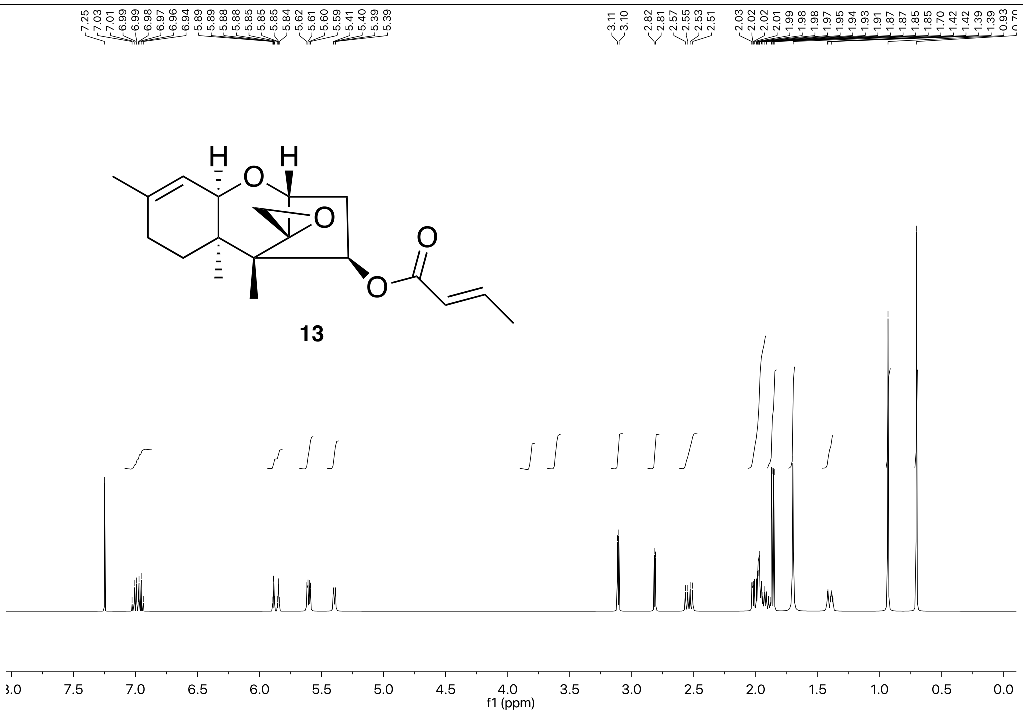

400 MHz ^{1}H NMR of compound **13** in $CDCl_3$

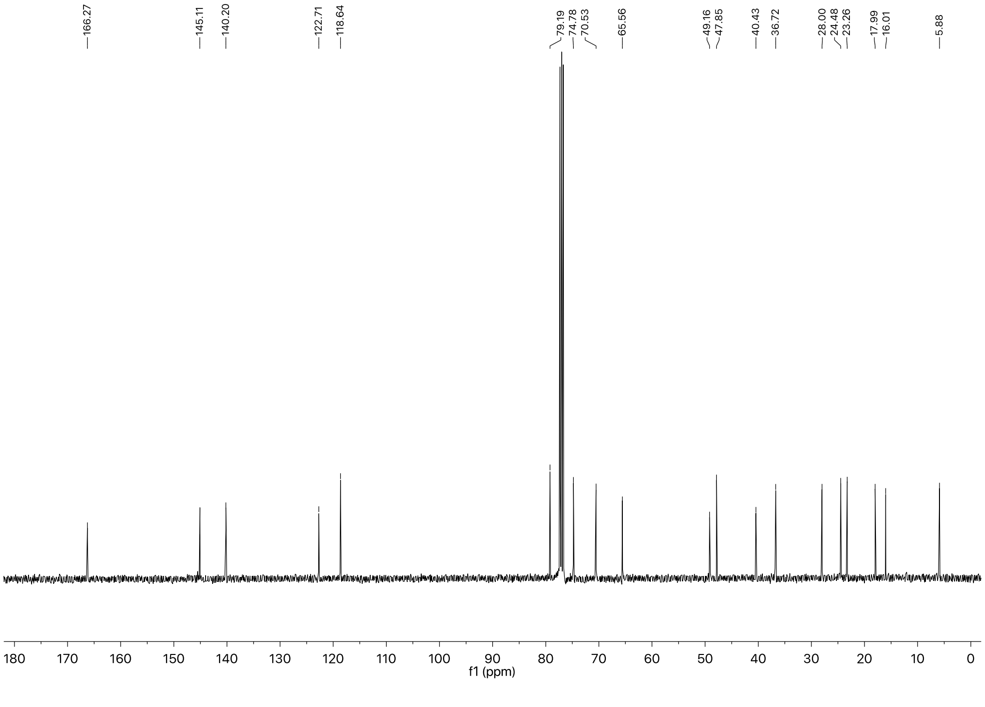

100 MHz ^{13}C NMR of compound **13** in $CDCl_3$

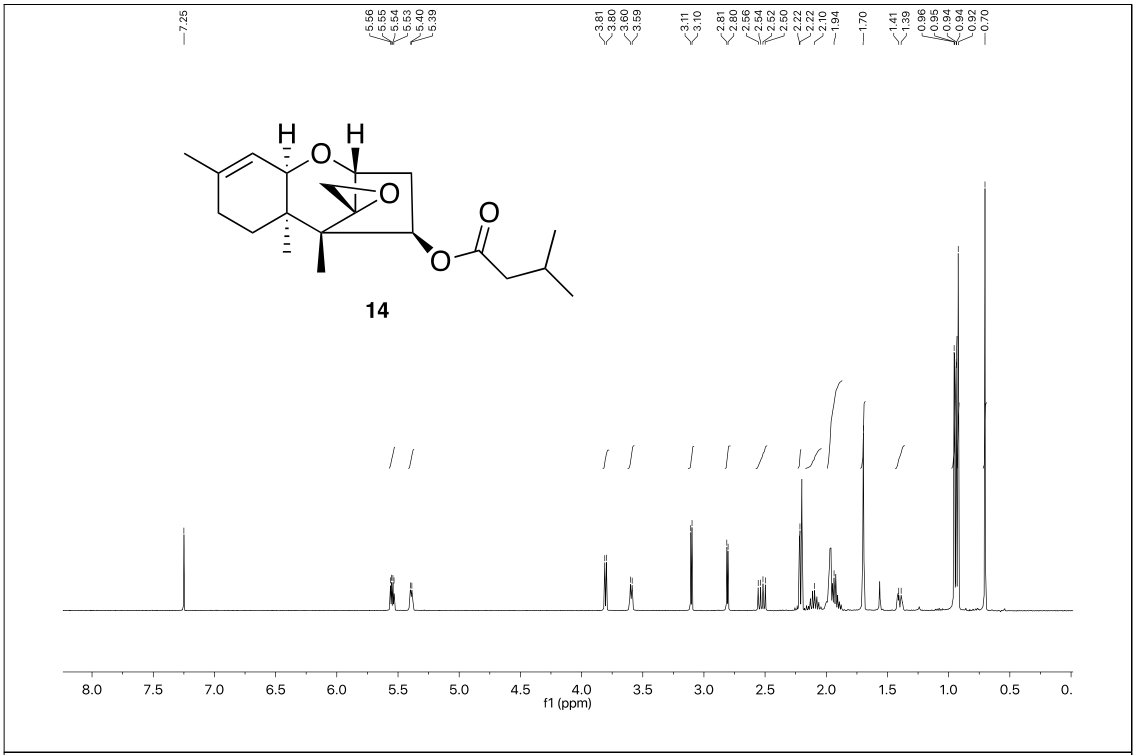

400 MHz ^{1}H NMR of compound **14** in $CDCl_3$

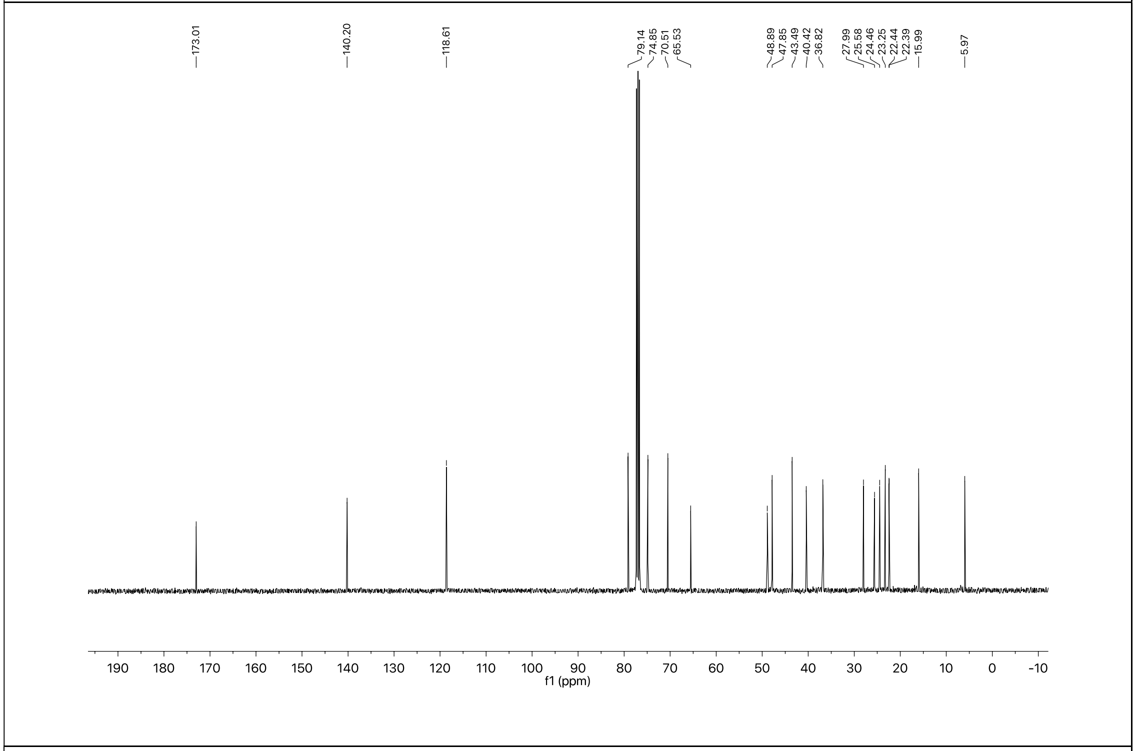

100 MHz ^{13}C NMR of compound **14** in $CDCl_3$

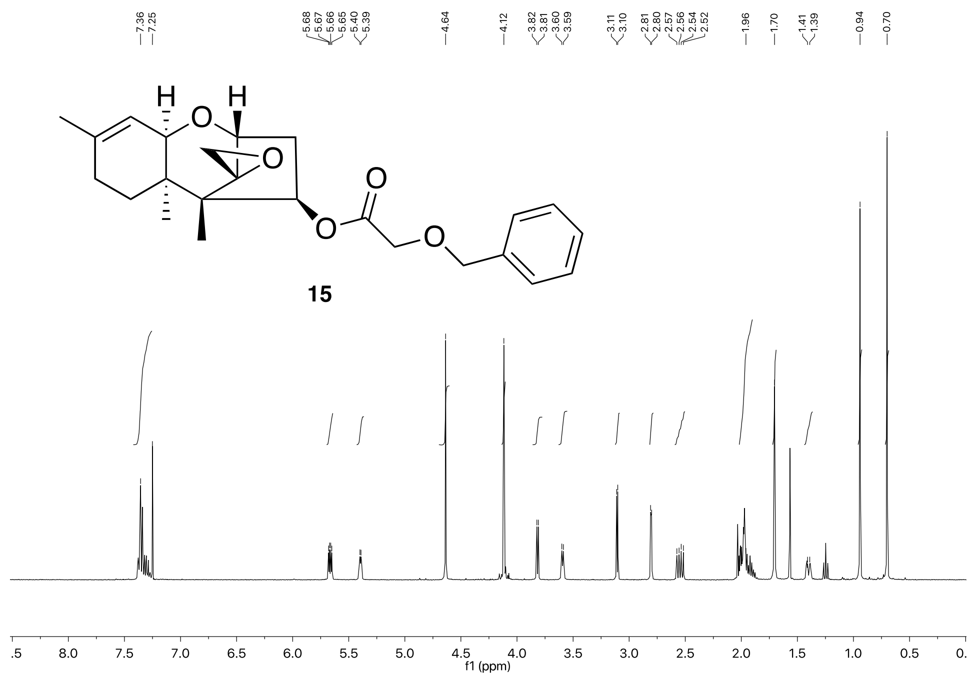

400 MHz ^{1}H NMR of compound **15** in $CDCl_3$

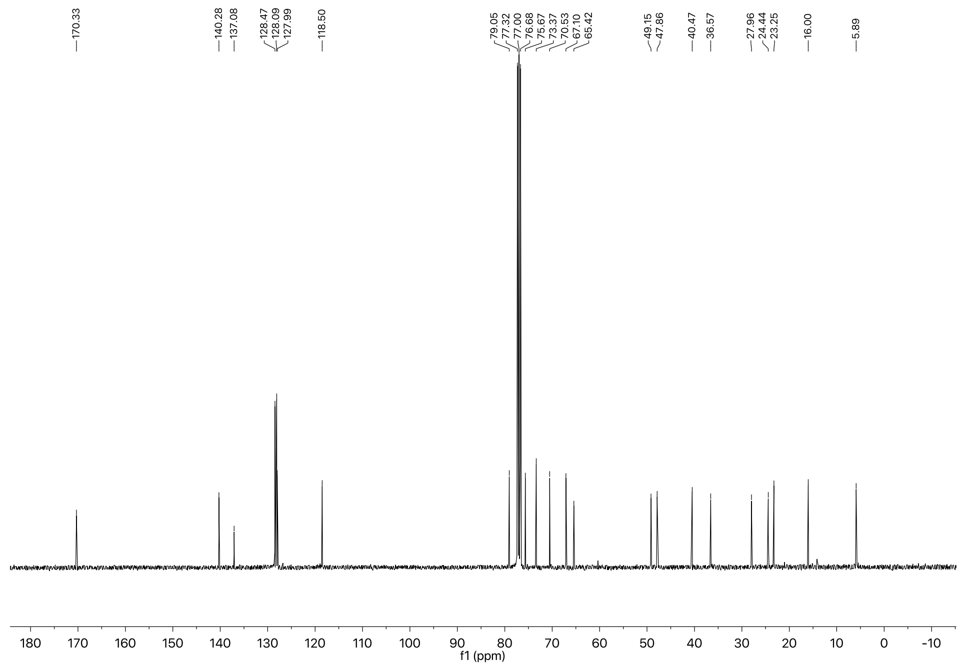

100 MHz ^{13}C NMR of compound **15** in $CDCl_3$

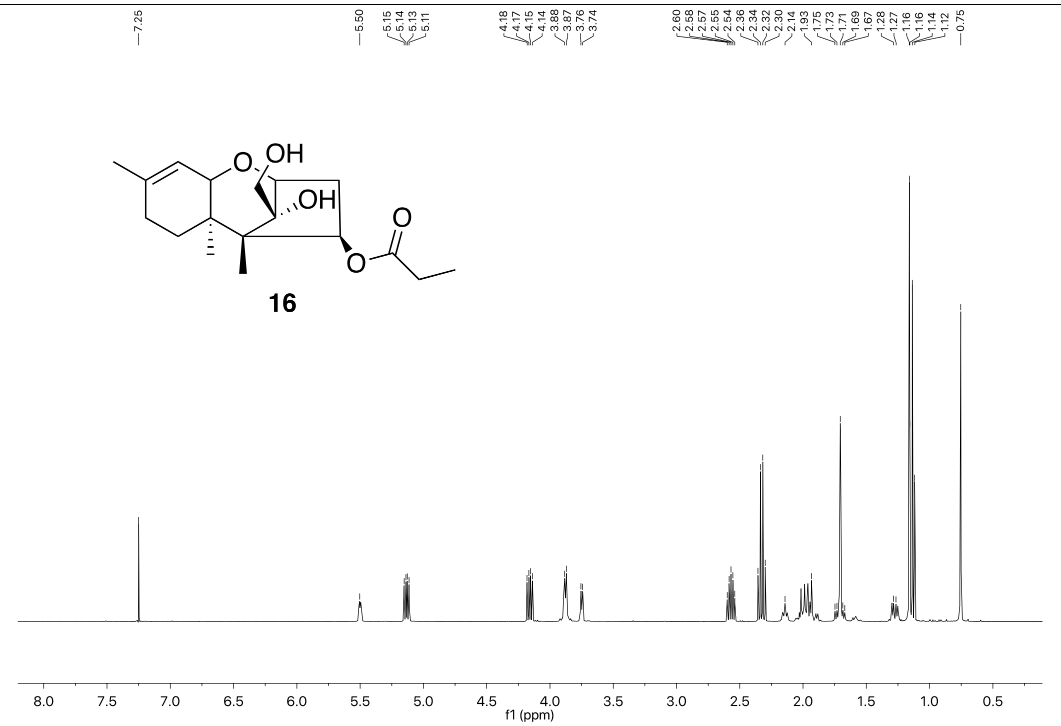

400 MHz ^{1}H NMR of compound **16** in $CDCl_3$

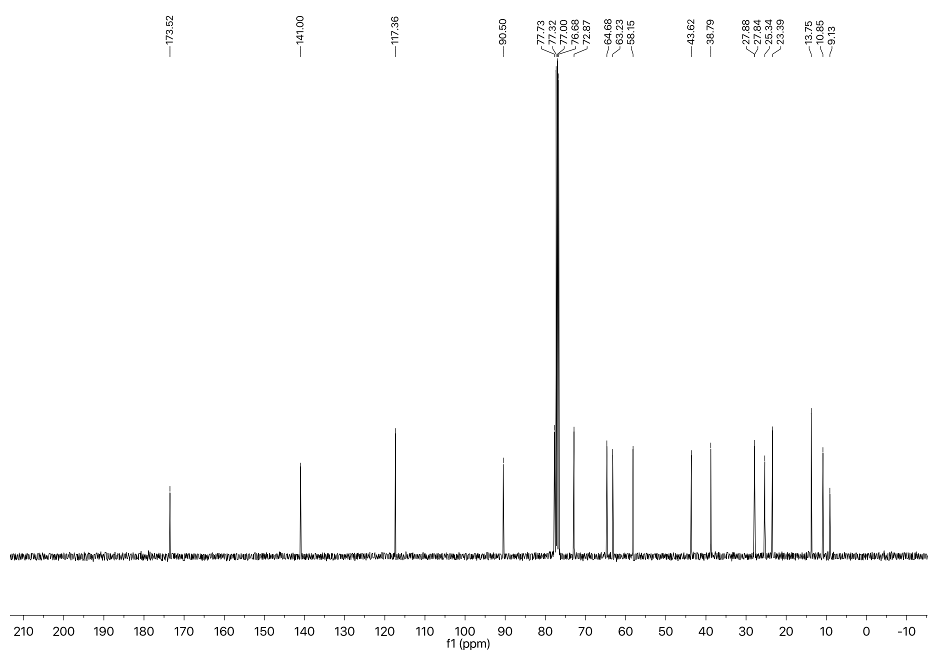

100 MHz ^{13}C NMR of compound **16** in $CDCl_3$
